# Genome and secretome analyses provide insights into keratin decomposition by novel proteases from the non-pathogenic fungus *Onygena corvina*

**DOI:** 10.1007/s00253-015-6805-9

**Published:** 2015-07-16

**Authors:** Yuhong Huang, Peter Kamp Busk, Florian-Alexander Herbst, Lene Lange

**Affiliations:** 1 Department of Chemistry and Bioscience, Aalborg University Copenhagen, 2450 Copenhagen, SV Denmark; 2 Center for Microbial Communities, Department of Chemistry and Bioscience, Aalborg University, Fredrik Bajers Vej 7H, 9220 Aalborg East, Denmark; 3 Department of Chemical and Biochemical Engineering, Technical University of Denmark, Building 229, 2800 Kgs. Lyngby, Denmark

**Keywords:** *Onygena corvina*, Keratinolytic protease, Enzyme blends, Genome, Peptide pattern recognition, Secretome

## Abstract

Poultry processing plants and slaughterhouses produce huge quantities of feathers and hair/bristle waste annually. These keratinaceous wastes are highly resistant to degradation. *Onygena corvina*, a non-pathogenic fungus, grows specifically on feathers, hooves, horn, and hair in nature. Hence, the proteases secreted by *O. corvina* are interesting in view of their potential relevance for industrial decomposition of keratinaceous wastes. We sequenced and assembled the genome of *O. corvina* and used a method called peptide pattern recognition to identify 73 different proteases. Comparative genome analysis of proteases in keratin-degrading and non-keratin-degrading fungi indicated that 18 putative secreted proteases from four protease families (M36, M35, M43, and S8) may be responsible for keratin decomposition. Twelve of the 18 predicted protease genes could be amplified from *O. corvina* grown on keratinaceous materials and were transformed into *Pichia pastoris*. One of the recombinant proteases belonging to the S8 family showed high keratin-degrading activity. Furthermore, 29 different proteases were identified by mass spectrometry in the culture broth of *O. corvina* grown on feathers and bristle. The culture broth was fractionated by ion exchange chromatography to isolate active fractions with five novel proteases belonging to three protease families (S8, M28, and M3). Enzyme blends composed of three of these five proteases, one from each family, showed high degree of degradation of keratin in vitro. A blend of novel proteases, such as those we discovered, could possibly find a use for degrading keratinaceous wastes and provide proteins, peptides, and amino acids as valuable ingredients for animal feed.

**Electronic supplementary material:**

The online version of this article (doi:10.1007/s00253-015-6805-9) contains supplementary material, which is available to authorized users.

## Introduction

Several million tons of feathers and hair/bristle wastes are generated annually by poultry processing industries and slaughterhouses (Swetlana and Jain 2010). The feathers and hair/bristle wastes are classified in category 3 animal by-products as low-risk materials for animals, the public, and environment (Korniłłowicz-Kowalska and Bohacz 2011). Nevertheless, disposal of feathers and hair/bristle waste is a challenge for the poultry processing industries and slaughterhouses because no easy method for removal of these materials exists. Keratin is the most abundant protein (at 90 %) present in hair/bristle and feathers where it has protective and structural functions (Korniłłowicz-Kowalska and Bohacz 2010). Keratin belongs to the intermediate filament proteins that are packed tightly either as a α-helix (α-keratin) or β-sheet (β-keratin) in a super coiled polypeptide chain with a high degree of cross-linking by disulfide bonds, hydrophobic interactions, and hydrogen bonds (Fraser and Parry 2008; Riffel et al. 2011). Thus, keratin is a very strong and insoluble protein with high resistance to mechanical stress and recalcitrance to common proteolytic enzymes like pepsin, trypsin, and papain (Riffel et al. 2011). So far, there is still no widely accepted, efficient, and commercialized process for breaking down animal-derived keratinaceous wastes or side stream products. Even though a large variety of bacteria, actinomycetes, and fungi are reported to be keratin degraders, commercial keratinases (Versazyme, Valkerase, and Prionzyme) are only available from *Bacillus licheniformis* PWD-1 strain based on KreA (Gupta et al. 2013) and Proteinase K is only from *Tritirachium album*. Nevertheless, fungal keratinolytic enzymes are increasingly recognized as an unexploited source, which could be further tested for possible industrial applications.

Proteases with potential to decompose keratin in natural materials such as feathers and hair/bristle have been found and described primarily for fungi that invade animal skin. These fungi belong to the group of human dermatophytes or pathogens such as *Arthroderma benhamiae*, *A. gypseum*, *A. otae*, *Microsporum canis*, *Trichophyton equinum*, *T. rubrum*, *T. tonsurans*, and *T. verrucosum* (Burmester et al. 2011; Martinez et al. 2012). Furthermore, multiple proteases have been associated with keratin degradation due to the fact that saprotrophic non-specific fungi such as *Trichoderma* sp., *Chrysosporium* sp., and *Aspergillus* sp. are also able to grow on keratinaceous materials (Avasn et al. 2011; Cao et al. 2008; Lopes et al. 2011). For safety reasons, the human pathogenic fungi are not acceptable as producers of enzyme blends at industrial scale. Nor are the human pathogens a preferred choice as sources of genes for recombinant expression of industrially relevant enzymes because the resulting enzymes may have strong inherent health risks. The keratinase activities of some of these fungi have been partly characterized without identification of the involved genes (Cheung and Maniotis 1973; Giudice et al. 2012). Some proteases of these fungi have also been purified or recombinantly expressed to investigate their role in infection but not for investigation of their potential use as industrial keratinases (Asahi et al. 1985; Brouta et al. 2002; Chen et al. 2010; Lee et al. 1987; Sriranganadane et al. 2011). Among the saprotrophic non-specific fungi, no single enzyme or blend of enzymes with strong keratin decomposition potentials has been found.

*Onygena corvina* (feather stalkball) and *Onygena equinea* (horn stalkball), both species of the fungal genus *Onygena* in the *Onygenaceae* family, can live as saprophytes on feathers, hooves, horn, and hair (Lange and Hora 1975). As non-pathogenic fungi, interesting enzymes of these species could be of relevance for industrial applications for converting keratin to feed protein, such as feather degradation and upgrade for use for food and feed ingredients and biotechnological applications. Many *Onygenales* are keratinophilic fungi that either behave as saprophytes on keratinaceous substrate or are pathogens of birds, mammals, and humans (Doveri et al. 2012). However, little is known about the keratinolytic potential of *O. corvina* and *O. equinea.* So far, only the ecological niche and keratinaceous substrate colonization of these species have been described.

**Fig. 1:**
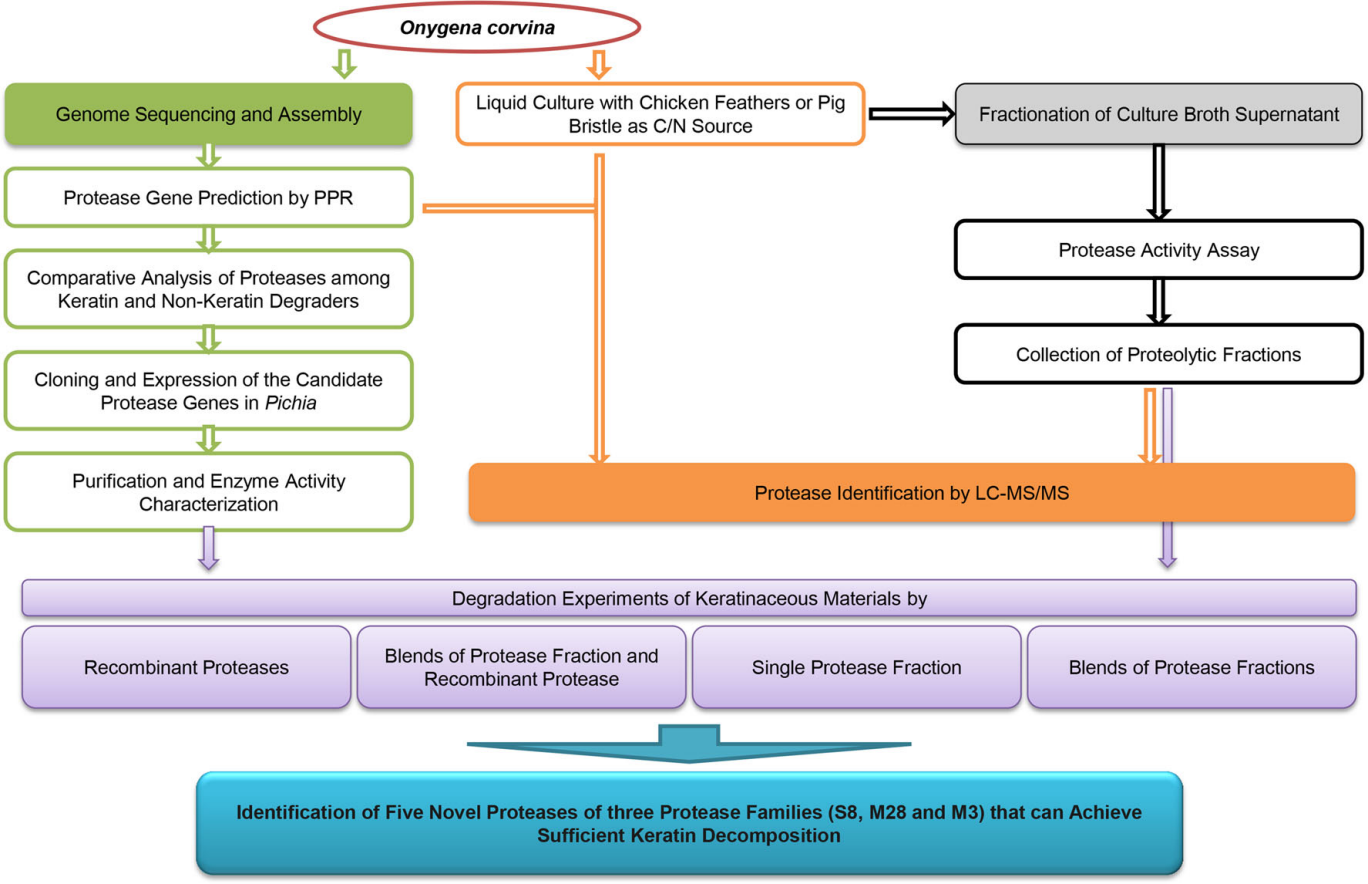
Flowchart showing genome (*green shading*) and secretome analysis (MS: *orange shading*, fractionation: *black shading*) of *O. corvina* keratinolytic proteases, further confirmed by the degradation of keratinaceous materials (*purple shading*)

The aim of the present study was to discover keratinolytic enzymes from the non-pathogenic fungus *O. corvina* according to genome and secretome analyses as shown in Fig. 1.

## Materials and methods

### Microorganism and growth conditions

*O. corvina* (strain number: CBS 281.48) was obtained from CBS-KNAW fungal biodiversity center (Centraalbureau voor Schimmelcultures, Utrecht, The Netherlands) and kept on potato dextrose agar plate at 4 °C. Subculturing was done once a month.

For protease production, a 4 mm^2^ square of *O. corvina* mycelium from a PDA plate was inoculated in a 50 ml minimal liquid culture medium containing 10 g/l chicken feathers/dog wool/20 g/l pig bristle; 2 g/l KH_2_PO_4_, 0.15 g/l MgSO_4_·7H_2_O, 0.3 g/l CaCl_2_, 3.3 g/l Tween 80, pH 8 and incubated at 25 °C on a rotary shaker (130 rpm) for 8 to 11 days (Lange et al. 2014).

Chicken feathers were obtained from Rose Poultry (Vinderup, Skovsgaard, Denmark) on 27 Nov. 2013. Pig bristle was obtained from Danish Crown (Bragesvej, Denmark) on 12 Nov. 2013. Dog wool was kindly provided by Signe Busk Lassen. Pretreated bristles and hooves (ground into particles approximately 1–2 mm in diameter) were obtained from Danish Crown (Bragesvej, Denmark) on 22 Mar. 2014. These materials (except pretreated bristles and hooves) were washed successively with tap water and distilled water. Then, they were cut into about 1 cm pieces and air dried. Before their application as sole carbon and nitrogen source in the minimal liquid medium, they were further dried in an oven at 50 °C to constant weight.

### Assay of protease activity with azocasein

Protease activity was assayed using azocasein substrate (Bach et al. 2011) by mixing 20 μl 1.5 % *w*/*v* azocasein (Sigma-Aldrich) suspensions in 2× McIlvaine buffer (pH 8) and 20 μl diluted enzyme in 1.5 ml tubes. The reactions were carried out at 50 °C for 1 h with constant agitation at 300 rpm using a TS-100 Thermo-Shaker, SC-20 (Biosan Ltd). After incubation, the reactions were stopped by adding 100 μl 0.4 M trichloroacetic acid (TCA) and incubating at 4 °C for 30 min. Then, the mixture was centrifuged at 16,000×*g* for 1 min to remove the substrate. One microliter supernatant was transferred to a microtiter plate containing 25 μl of 1.8 M NaOH. Absorbance was read at 405 nm using a plate reader. A control was prepared using 20 μl 1.5 % *w*/*v* azocasein suspension in the same buffer to which 100 μl 0.4 M TCA was added before adding 20 μl enzyme solution. The mixture was incubated at 50 °C for 1 h and treated in the same way as the sample.

One arbitrary unit (U) of protease activity was defined as the amount of enzyme causing 0.01 absorbance increase between the sample and control at 405 nm under the assay conditions.

### Assay of protease activity with pig bristle

Enzymes/blends (0.05/0.025 ml) were incubated with 4 mg pig bristle (cut into approximately 5 mm pieces) or pretreated bristles and hooves (ground into particles approximately 1–2 mm in diameter) in 0.2 ml 2× McIlvaine buffer (pH 8) in 1.5 ml tubes. Assays were run at 40 °C for 24 h or 4 days with constant agitation at 1000 rpm. The initial and final soluble protein in supernatant was measured at 280 nm by NanoDrop 1000 (Thermo Scientific) before and after incubation. The increased soluble protein was calculated as the difference between the final and initial soluble protein. As a control, culture supernatant was replaced by 2× McIlvaine buffer (pH 8) and treated in the same way as the sample. The commercial keratinolytic proteases esperase (5860, Sigma-Aldrich), Alcalase (4860, Sigma-Aldrich), and Savinase (3111, Sigma-Aldrich) were also included in the assay for comparison. Purified bovine serum albumin (BSA) (10 mg/ml) was serially diluted to 0, 0.1, 0.2, 0.4, 0.6, 0.8, 1.0, 1.2, 1.4, 1.6, 1.8, 2.0 mg/ml in 2× McIlvaine buffer (pH 8). A standard curve was generated by measuring diluted BSA absorbance at 280 nm. The soluble protein before and after pig bristle degradation was calculated according to the BSA standard curve. The degree of pig bristle degradation was calculated using the equation. The degree of degradation was calibrated to negative control.

$$ \mathrm{Degree}\;\mathrm{of}\;\mathrm{degradation}\left(\%\right)=\mathrm{increased}\;\mathrm{soluble}\;\mathrm{protein}\left(\mathrm{mg}\right)/\mathrm{initial}\;\mathrm{pig}\;\mathrm{bristle}\;\mathrm{weight}\left(\mathrm{mg}\right)\times 100 $$

### Assay of purified recombinant protease with fluorescein-labeled casein

Purified protease activities were analyzed with fluorescein-labeled casein (Pierce Fluorescent Protease Assay Kit, 23266, Thermo Scientific) by fluorescence resonance energy transfer (FRET) on a Corbett Rotor Gene 6000 (Corbett Research). The fluorescein excitation and emission filters were 470 and 510 nm, respectively. Fluorescein isothiocyanate-casein (FTC-casein) working reagent and trypsin standard were prepared as described in the manufacturer’s manual. First, 20 μl sample or standard was added to QIAsafe DNA Tubes (SAP 1055470, Qiagen). Next, 20 μl FTC-casein working reagent was added to the tubes, and the mixture was incubated at 30 °C for 60–90 min. The protease activity was measured as the increase in real-time fluorescence over the reaction time.

### Genomic DNA extraction

*O. corvina* was cultured in YPD liquid medium at 25 °C and 130 rpm for 3 days. The mycelium was filtered on a nylon mesh and ground with a mortar and pestle in liquid nitrogen. Genomic DNA was extracted with the DNeasy plant mini kit (69104, Qiagen) following the manufacturer’s protocol. The quantity and quality of the genomic DNA was measured on a NanoDrop 1000 (Thermo Scientific) and by electrophoresis on 1 % agarose gel.

### De novo draft genome assembly

The genome of *O. corvina* was sequenced on an Illumina Hiseq 2000 in one multiplexed lane as paired-end libraries with Truseq chemistry by AROS Applied Biotechnology A/S, Denmark. Based on the estimated genome size, the sequence coverage was 370 times. The raw sequences were filtered for residual adapter sequences and trimmed with AdapterRemoval v1.5.2 (Lindgreen 2012) and Seqtk (Heng 2012). The clean sequences were de novo assembled with CLC Genomics Workbench 6.0.5. Assembly statistics were calculated with the Assemblathon script (Earl et al. 2011).

### Gene annotation by homology to peptide patterns (Hotpep)

Gene annotation by Hotpep was done as previously described (Busk and Lange 2013). The predicted protein sequences of the hits were confirmed by BLAST. Full-length genes of the validated proteases were obtained by assembling the related contigs with CLC Main Workbench 6, and the open reading frame (ORF) was predicted by Augustus (Stanke et al. 2004). The ORFs were further validated by BLAST.

### Protease comparison

The genomes of eight keratin-degrading fungi and four non-keratin-degrading fungi were downloaded from GenBank (Table 1).

**Table 1:** List of fungi whose genomes were mined for protease encoding genes

Fungus	GenBank assembly accession	Keratin degrader
*Arthroderma otae*	GCA_000151145.1	Yes
*A. gypseum*	GCA_000150975.1	Yes
*Batrachochytrium dendrobatidis*	GCA_000149865.1	Yes
*Coccidioides posadasii*	GCA_000150245.1	Yes
*C. immitis*	GCA_000149335.1	Yes
*Onygena corvina*	New assembly in this study	Yes
*Trichophyton rubrum*	GCA_000151425.1	Yes
*T. tonsurans*	GCA_000151455.1	Yes
*T. verrucosum*	GCF_000151505.1	Yes
*Homoloaphlyctis polyrhiza*	GCA_000235945.1	No
*Saccharomyces cerevisiae*	GCA_000146045.2	No
*Talaromyces stipitatus*	GCA_000003125.1	No
*Wickerhamomyces anomalus*	GCA_000147375.2	No

The number of proteases of each Merops family found by PPR in the genomes of *O. corvina* and the eight keratin-degrading fungi were compared to the number of proteases of each Merops family found in the genomes of the four non-keratin-degrading fungi.

### Signal peptide prediction and phylogenetic analysis

Signal peptides were predicted with SignalP 4.1 (Petersen et al. 2011).

The phylogenetic analysis was performed as follows: S8 protease family protein sequences were downloaded from NCBI; then, the protease sequences were aligned using Clustal X (Thompson et al. 1997), and phylogenetic tree was generated using the neighbor-joining algorithm in MEGA ver5.0 (Tamura et al. 2011).

### RNA extraction

*O. corvina* was grown on feathers, pig bristle, or dog wool for 7 days after which around 100 mg of mycelium together with keratinaceous materials were thoroughly disrupted in lysis buffer by 3 × 20 s pulses in a FastPrep-24 homogenizer (MP Bio). Total RNA was extracted with the RNeasy plant mini kit (74904, Qiagen). Genomic DNA was removed by treatment with DNase I (RNase-free) (M0303L, New England Biolabs Inc.). The quality and quantity of the RNA were measured by NanoDrop 1000 (Thermo Scientific) and electrophoresis on 1 % agarose gel.

### cDNA synthesis

Reverse transcription of total RNA was performed according to Madsen et al. (2009). The reaction contained the total RNA (200 ng), 4 μl mixed primers (oligodT primer/random hexamer primer = 1:3 (0.5 μg/μl)), 16 μl 5× ImPromII buffer, 4 μl dNTP mix (10 mM each), 8 μl 25 mM MgCl_2_, and 4 μl ImpromII (Promega). The single-stranded DNA product was stored at −20 °C.

### Amplification, cloning, and expression of putative protease genes

Eighteen predicted protease genes were amplified from cDNA made of RNA extracted from *O. corvina* grown on feathers, pig bristle, or dog wool with specific primers with a His-tag-encoding sequence added at the 5′-end of the reverse primer (Table S1). The PCR reaction mixtures contained 1.5 μl diluted cDNA, 10 μl 5× Phusion® HF buffer, 1 μl 10 mM dNTP (Fermentas), 2.5 μl 10 μM each primer, and 1 U Phusion High-Fidelity DNA Polymerase (M0530S, New England Biolabs Inc.). The PCR reaction was performed in Biometra Thermocyclers T3000. The initial denaturation step (98 °C, 30 s) was followed by 30 cycles of denaturation (98 °C, 10 s), annealing (see annealing temperature for each gene in Table S1, 30 s) and elongation (72 °C, 30 s per kb), and an additional elongation step (72 °C, 10 min) after the final cycle. The PCR products were purified with the GeneJET Gel Extraction and DNA Cleanup Mini Kit (K0831, Thermo Scientific) and digested with the restriction enzymes (New England Biolabs Inc.) as indicated in Table S1.

The vector pPinkα-HC (PichiaPink Expression System, Invitrogen) was digested with *Stu*I and *Fse*I restriction enzymes and purified. The digested PCR products were inserted into pPinkα-HC vector with T4 DNA ligase (EL0011, Thermo Scientific). The recombinant plasmids were transformed to *Escherichia coli* DH5α. Positive clones were selected on LB plates with 100 μg/ml ampicillin and identified by colony PCR and sequencing. About 5–10 μg vector with protease genes was linearized with *Spe*I (except gene 3998, which was linearized by *Eco*NI) and transformed into PichiaPink Strain 4 (Invitrogen) by electroporation according to the manufacturer’s manual. After incubation in YPDS medium for 2 h at 30 °C without shaking, positive clones were selected on PAD plates (A11156, PichiaPink Media Kit, Invitrogen) by incubation at 30 °C for 3–7 days.

A single white clone was chosen for each recombinant protease gene. Expression of recombinant proteases was performed by growing the recombinant strains in 200 ml BMMY medium (10 g/l yeast extract, 20 g/l peptone, 13.4 g/l yeast nitrogen base, 4 × 10^−5^ g/l biotin, 5 ml/l methanol, and 100 mM potassium phosphate buffer, pH 6.0) at 28 °C for 4 days with methanol as both the sole carbon source and the inducer (PichiaPink Expression System manual). The supernatant was harvested after 4-day incubation by centrifugation at 1500×*g* for 5 min at 4 °C and filtration through a 0.2 μm filter (Minisart NML Syringe Filters 16534, Sartorius) and was stored at −80 °C.

### Purification of expressed proteases

The His-tagged proteases were purified by fast protein liquid chromatography (FPLC) (ÄKTA Purifier) by the UNICORN method on a 1 ml HisTrap FF crude affinity column (11-0004-58, GE Healthcare) as previously described (Huang et al. 2014).

### Protein determination of purified recombinant proteases

The protein concentration of purified recombinant enzyme was estimated using absorbance at 280 nm and the computed molar extinction coefficient of the protein sequence.

### Precipitation of proteins in culture broth supernatant of *O. corvina* for MS analysis

Culture broth produced as described earlier under microorganisms and growth conditions (Lange et al. 2014) was harvested by centrifugation at 10,000×*g* for 15 min at 4 °C. The supernatant was filtered (0.2 μm, Minisart). The secreted proteins were precipitated by incubating 30 ml filtered supernatant with freshly prepared 3 g crystalline TCA (final concentration 10 % *w*/*v*) and kept at −20 °C in a freezer overnight (Hempel et al. 2011). The precipitate was pelleted by thawing and centrifugation at 10,000×*g* for 30 min at 4 °C. The protein pellet was washed three times with 1 ml ice-cold acetone and subsequently centrifuged at 14,000×*g* for 5 min at 4 °C. Finally, the protein pellet was air dried.

### Ion exchange chromatography of wild-type proteases

Culture broth supernatant was harvested by centrifugation at 10,000×*g* for 10 min at 4 °C. The supernatant was filtered (0.2 μm, Minisart) and fractionated using two separate methods: (1) cation exchange (5 ml HiTrap SP column, 50 mM citrate buffer, pH 3.86) and (2) anion exchange (1 ml HiTrap Q column, 20 mM Tris buffer, pH 8.6). In both cases, 50 ml of filtered culture broth supernatant were applied to the column, and a NaCl gradient from 0 to 1 M NaCl was applied to elude the bound protein.

Two hundred microliter anion exchange fractions and 400 μl cation exchange fractions were heated to 90 °C for 15 min and dried. Protease identification by in-solution digestion and LC-MS/MS analysis was performed as below.

### Generation of tryptic peptides

The protein pellet was solubilized in digestion buffer (1 % sodium deoxycholate, 50 mM triethylammonium bicarbonate, pH 8.0) and heated to 99 °C for 5–10 min. The sample was kept at 37 °C, and 1 μg Tris (2-carboxyethyl) phosphine was added per 25 μg sample protein and incubated for 30 min at 60 °C. Next, 1 μg iodoacetamide (from a 2.5 μg/μl iodoacetamide stock solution in water) was added per 10 μg sample protein followed by incubation for 20 min at 37 °C in the dark. Then, the sample was digested by the addition of 1 μg trypsin (from a 0.1 μg/μl trypsin stock solution) per 50 μg sample protein and incubated overnight at 37 °C. The reaction was stopped and the deoxycholate precipitated by the addition of formic acid to a final concentration of 2 %, mixing and incubation at room temperature for 5 min. The sample was centrifuged at 13,000×*g* for 20 min at 4 °C, and the supernatant was recovered and dried by vacuum centrifugation. All tryptic peptide preparations were purified using StageTips packed with Poros Oligo R3 material (Applied Biosystems) on top of two C18 disks (3 M, Bioanalytical Technologies) as previously described (Rappsilber et al. 2003, 2007). Peptides were eluted with 70 % (*v*/*v*) acetonitrile and dried.

### Analysis of proteins by LC-MS/MS

Peptides were reconstituted in 0.1 % trifluoroacetic acid/2 % acetonitrile solution. Eight microliter of each sample was injected by autosampler and concentrated on a trapping column (Pepmap100, C18, 100 μm × 2 cm, 5 μm, Thermo Fisher Scientific) with water containing 0.1 % formic acid and 2 % ACN at a flow rate of 4 μl/min. After 10 min, the peptides were eluted into a separation column (PepmapRSLC, C18, 75 μm × 50 cm, 2 μm, Thermo Fisher Scientific). Chromatography was performed with 0.1 % formic acid in solvent A (100 % water) and B (100 % acetonitrile). Two linear gradients with solvent B were run one after another. First from 4 to 12 % over 5 min, then from 12 to 30 % over 30 min followed by a final step gradient to 90 % solvent B, which was maintained for 5 min using a nano-high-pressure liquid chromatography system (Ultimate 3000 UHPLC, Thermo Fisher Scientific). Ionized peptides were measured and fragmented by a Q Exactive mass spectrometer (Thermo Fisher Scientific). For an unbiased analysis, continuous scanning of eluted peptide ions was carried out between 400 and 12,000 *m*/*z*, automatically switching to MS/MS higher energy collisional dissociation (HCD) mode and 12 MS/MS events per survey scan. For MS/MS HCD measurements, a dynamic precursor exclusion of 30 s, peptide match, and an apex trigger of 2 to 10 s were enabled.

### MS data analysis

Protein identification was done with the open-source software MaxQuant (v. 1.4.1.2) (Cox and Mann 2008). The label-free quantification (LFQ) algorithm (Cox et al. 2014) and the *match between runs* feature were activated. Carbamidomethylation of cysteines was defined as fixed modification and oxidation of methionines as well as N-terminal acetylation as variable modification. The remaining settings were kept on default. This includes a maximum peptide and protein false discovery rate of 1 % and a minimum of two peptides for LFQ calculation. ORFs within the *O. corvina* genome were translated into amino acid sequences and kept if they consisted of at least 100 amino acids. This six-frame database was created and used as a search database in MaxQuant. The mean LFQ per protein was calculated if a protein was quantified in at least two out of three biological replicates. For comparison of relative changes, the LFQ ratios between conditions were formed and log_2_ transformed. Statistical significances of abundance changes were assessed by *t* test (two-tailed, heteroscedastic). Batch CD search (Marchler-Bauer et al. 2011) was used to search for conserved domains and annotation of identified protein.

### Data availability

This Whole Genome Shotgun project has been deposited at GenBank under the accession JWPT00000000. The version described in this paper is version JWPT01000000. The predicted protease nucleotide sequences have been deposited at GenBank under accession numbers KP290810-KP290882 (Tables 2, 3, and S2).

**Table 2:** Amplification results of candidate protease genes from cDNA template

Gene ID	Accession	Family/subgroup	Chicken feathers	Pig bristle	Dog wool
7508	KP290862	S8/SUB1	+	−	−
6266	KP290870	S8/SUB2	−	−	−
6877	KP290860	S8/SUB3	+	+	+
8702	KP290865	S8/SUB3	−	+	+
8545	KP290864	S8/SUB7	−	+	+
11652	KP290866	S8/SUB7D	−	+	+
11813	KP290867	S8/SUB8	+	+	+
7122	KP290861	S8/SUB9	+	+	+
14354	KP290868	S8/SUB11	+	+	+
6582	KP290859	S8/SUB12	−	−	−
8301^a^	KP290810	M35/NPIIB	−	−	−
7758	KP290811	M35/NPIID	+	+	+
11002	KP290815	M36/MEP1	−	−	−
12526	KP290812	M36/MEP2	−	−	−
8814	KP290813	M36/MEP3	−	−	−
3998	KP290814	M36/MEP4	+	+	+
3705	KP290816	M43/MEP6	−	−	+
6296	KP290817	M43/MEP8	+	+	+

The related RNA was extracted from *O. corvina* when it had grown on chicken feathers, pig bristle, or dog wool. “+” indicates that the protease coding sequence can be amplified from cDNA template

^a^The cDNA sequence of gene 8301 was synthesized and cloned into pUC57 by GenScript (USA)

**Table 3:** Protease compositions of fractions A10, A11, C15, and C20 with strong keratin decomposition potential

Gene ID	Accession	Family	Annotation	A10	A11	C15	C20
14354	KP290868	S8	Serine protease	+	+	−	−
5775	KP290872	M1	Aminopeptidase 2	−	+	−	−
11652	KP290866	S8	Alkaline serine protease	−	−	−	+
2945	KP290851	M14	Metallocarboxypeptidases	+	−	−	−
6423	KP290880	M28	Leucine aminopeptidase	+	+	+	+
8393	KP290873	M3	Metallopeptidase	+	+	+	−
6844	KP290843	M28	Peptidase	+	+	−	−
8832	KP290855	M20	Peptidase	+	+	−	−
10291	KP290818	S28	Serine carboxypeptidase	+	−	−	−
11813	KP290867	S8	Serine proteinase	+	+	−	−
8472	KP290881	–	Aspartate aminotransferase	+	+	−	−
7142	KP290852	M14	Carboxypeptidase	+	+	−	−
9005	KP290875	S9	Dipeptidyl peptidase 4	+	−	−	−
8025	KP290838	M28	Leucyl aminopeptidase	−	−	−	+
13394	KP290874	M49	Dipeptidyl peptidase 3	+	+	−	−
6877	KP290860	S8	Serine protease	+	+	+	+
3998	KP290814	M36	Metallopeptidase	+	+	−	−
4005	KP290877	S10	Carboxypeptidase	+	−	−	−
Total proteases				15	12	3	4

## Results

### Genomic analysis of protease genes in *O. corvina*

#### Genome sequencing and de novo assembly

After Illumina Hiseq 2000 sequencing, we obtained 81,538,322 paired-end 100 bp reads. The raw reads were cleaned, pooled together, and de novo assembled with the CLC Genomics Workbench. The result yielded 992 contigs with length ≥198 bp. The average contig length was 22,096 bp, the maximum was 933,412 bp, 98.9 % of the reads were matched successfully, and 91.3 % of the reads were in pairs (Tables S3 and S4). The *N*_50_ was 260 kb which is considerably higher than the assemblies of the dermatophytic fungi *T. rubrum*, *T. tonsurans*, *T. equinum*, *M. canis*, and *M. gypseum*, with *N*_50_ ranging from 27 to 146 kb (Martinez et al. 2012). The GC content of the *O. corvina* genome is 48 %. The genome size of *O. corvina* is approx. 22 Mb. It is very close to the genome size of other *Onygenales* (Martinez et al. 2012; Muszewska et al. 2011).

#### Prediction of protease genes in *O. corvina* genome by PPR

**Fig. 2:**
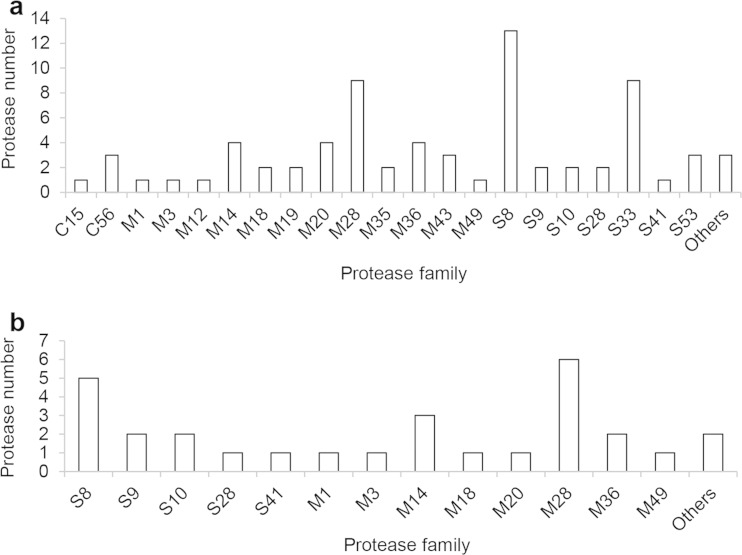
Spectrum of protease families in *O. corvina* genome (**a**), with number of protease genes from each family indicated; mass spectrometry data (**b**) identifying protease genes found in *O. corvina* secretome when grown on chicken feathers or pig bristle

Next, we applied PPR to find protease genes in the *O. corvina* genome. The hits were confirmed by blast, and full-length ORFs were predicted by Augustus. This led to identification of 73 putative proteases (Table S2). Clan information was obtained based on the Merops database, and the families were classified using the conserved domains. Most of the proteases are metalloproteases (M1, M3, M12, M14, M18, M19, M20, M28, M35, M36, M43, and M49), serine proteases (S8, S9, S10, S28, S33, S49, and S53), and cysteine proteases (C15 and C56). A majority of proteases belong to the families S8, S33, and M28 (Fig. 2a). A signal peptide was predicted in 12 of the 13 S8 proteases and in most of M28 proteases, which suggests that they are secreted proteins, whereas no signal peptide was predicted in any of S33 family proteases.

**Fig. 3:**
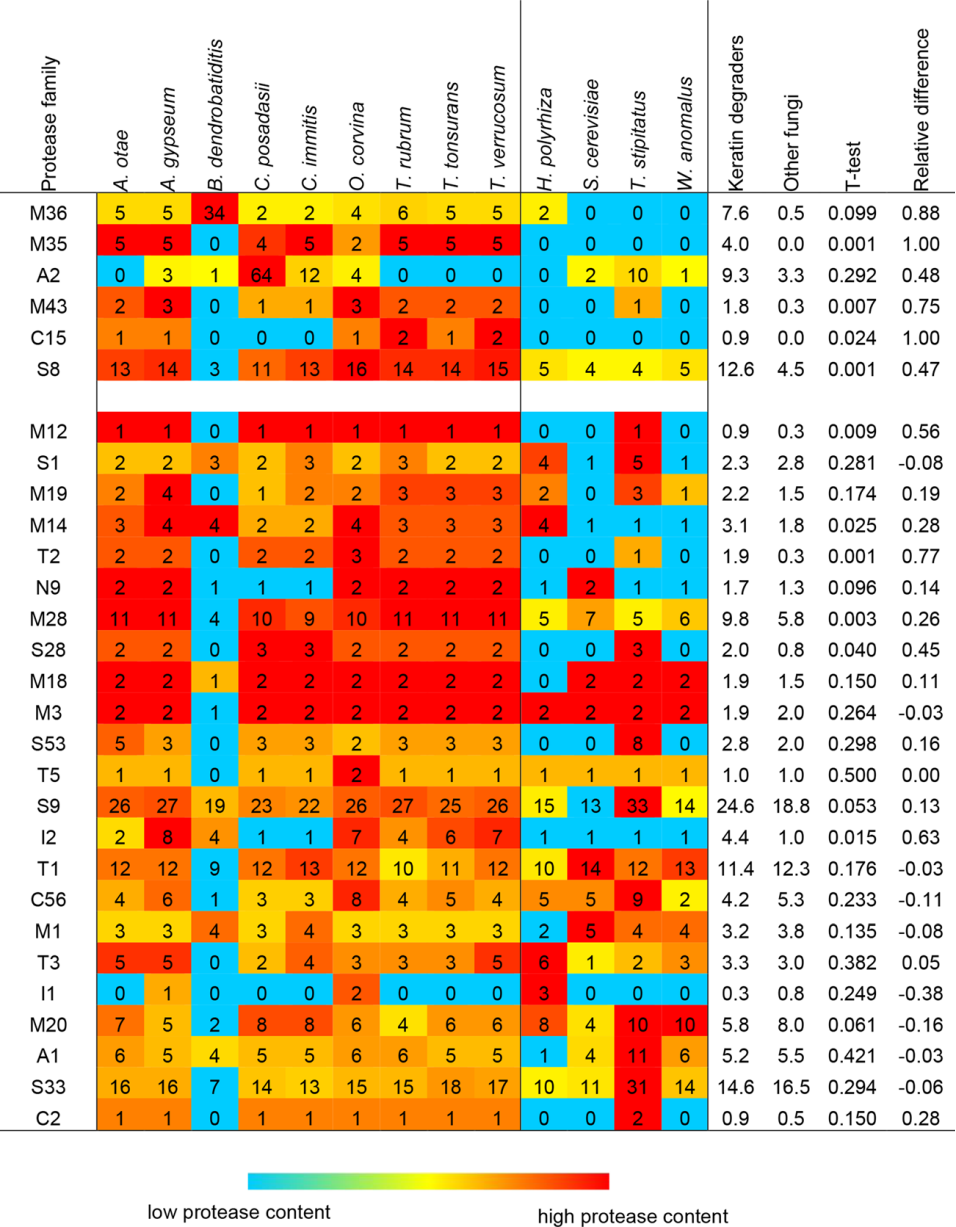
Comparative analysis of protease repertoire in a selection of keratin-degrading fungi (*left*: *Arthroderma otae*, *A. gypseum*, *Batrachochytrium dendrobatidis*, *Coccidioides posadasii*, *C. immitis*, *Onygena corvina*, *Trichophyton rubrum*, *T. tonsurans*, *T. verrucosum*) and non-keratin-degrading fungi (*right*: *Homoloaphlyctis polyrhiza*, *Saccharomyces cerevisiae*, *Talaromyces stipitatus*, *Wickerhamomyces anomalus*), used as basis for selecting keratinolytic protease genes characteristic for keratin decomposers. Mean number of proteases in genomes of keratin-degrading and non-keratin-degrading fungi was also calculated

Comparison of the number of proteases of each Merops family found in the genome of *O. corvina* and eight keratin-degrading fungi with those found in the genomes of four non-keratin-degrading fungi showed that among others, M36, M35, M43, C15, and S8 families are more abundant in keratin degraders than in non-keratin degraders (Fig. 3). Eight proteases from M36, M35, and M43 families in *O. corvina* genome were also shown to have signal peptides. Among 13 serine proteases (S8) identified in *O. corvina* genome, ten proteases were found to be subtilisin-like serine proteases with high diversity (Fig. S1).

#### Cloning and expression of related keratinolytic protease genes

Eighteen candidate protease genes were selected for PCR amplification with specific primers (Table S1) from cDNA template. The related RNA was extracted from *O. corvina* grown on chicken feathers (rich in β-keratin), pig bristle, or dog wool (rich in α-keratin). Twelve of 18 genes could be amplified, and most of these were from cDNA made from *O. corvina* growing on α-keratin-containing pig bristle or dog wool (Table 2). These 13 genes (including the synthetic gene for 8301 gene model) were expressed in PichiaPink strain 4. The recombinant proteases were purified, and protease activities were semiquantitatively analyzed using FTC-casein. Comparisons of the fluorescence curves showed the highest protease activity for protease 6877 (accession number: KP290860, S8), which was followed by protease 3998 (accession number: KP290814, M36) (Fig. S2). SDS-PAGE results showed that proteases 6877 and 3998 were comprised only one major band at the expected size in gel (Fig. S3). Unfortunately, the other purified recombinant proteases had very low protease activity (Fig. S2).

**Fig. 4:**
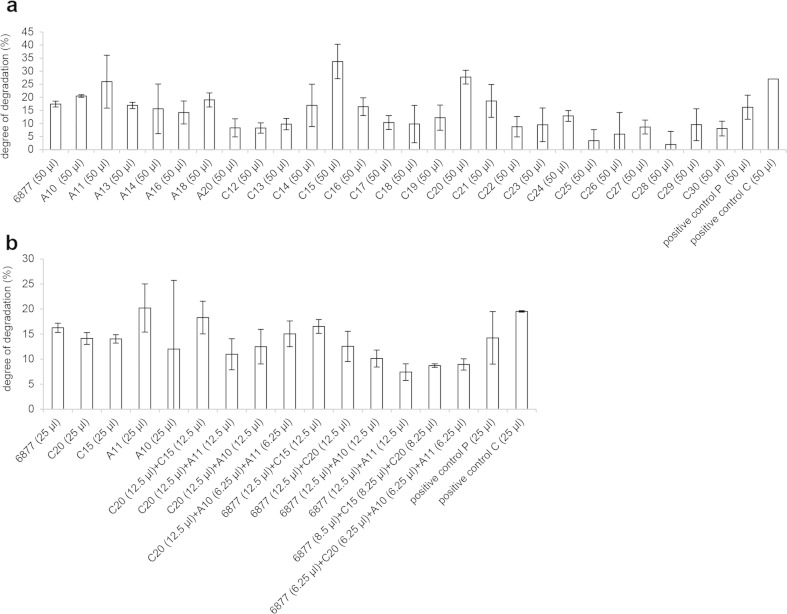
Degradation of keratinaceous materials by culture broth, fractions of culture broth, purified recombinant protease, and enzyme blends. **a** Treatment with different fractions of culture broth. **b** Treatment with blends of different fractions of culture broth and recombinant protease 6877. The degree of degradation was calibrated to negative control. Fraction labels beginning with A and C refer to anion (*A*) and cation (*C*) exchanged fractions, respectively. 6877 is the purified recombinant protease expressed in *P. pastoris*. Positive controls: culture broth supernatant from *O. corvina* grown for 11 days on fermentation medium with pig bristle (*P*) and chicken feathers (*C*), respectively. Negative control: 2× McIlvaine buffer (pH 8)

The recombinant protease with the highest activity (6877) was tested for ability to degrade pig bristle. The results showed that 50 and 25 μl, respectively, of this protease degrade 17 and 16 % of pig bristle in 24 h at 40 °C (Fig. 4a, b). The blend made by mixing 6877 and 3998 was also tested for pig bristle degradation but did not lead to an increased level of degradation (data not shown).

### Proteomic analysis of protease genes in *O. corvina*

#### MS analysis of protease composition in *O. corvina* secretome

MS data from *O. corvina* secretome was analyzed to directly identify additional novel keratinolytic proteases present in the culture broth, which was shown to decompose α- and β-keratin (Lange et al. 2014).

MS analysis identified 29 different proteases in the culture broth from *O. corvina* grown on chicken feathers or pig bristle, which correspond to around 40 % of the proteases in the *O. corvina* genome (Table S5). These secreted proteases were mainly metalloproteases (M1, M3, M14, M18, M20, M28, M36, and M49) and serine proteases (S8, S9, S10, S28, and S41). More M28 and S8 family proteases than the other protease families were found in the secreted proteases (Fig. 2b).

#### Ion exchange fractionation of culture broth supernatant of *O. corvina*

The *O. corvina* culture broth supernatant was fractionated by ion exchange chromatography to identify specific proteases involved in keratin degradation. Fractionation by cation exchange resulted in several fractions with strong protease activity (Table S6). Fractionation by anion exchange gave fractions with lower activity compared to cation exchange fractionation (Table S7). Fractions with protease activity were in-solution digested and analyzed by LC-MS/MS to further identify protease composition. When compared with the 29 proteases found in secretome analysis, only six of these proteases (peptidase (S41), putative secreted metalloprotease (M36), carboxypeptidase (M14), peptidase (M28), ornithine aminotransferase, dipeptidyl-peptidase (S9)) were not identified by MS as they were not fractionated in ion exchange chromatography and were eluted as a not bound fraction. The cation exchanged fractions (C) resulted in better separation of the proteases than the anion exchanged fractions (A). Whereas most cation exchanged fractions contained two to five proteases, the anion exchange fraction A13 had 18 proteases and the A10 and A14 fractions had 15 and 14 proteases, respectively (Table S8).

#### Pig bristle degraded by fractions of culture broth

The protease-containing fractions of culture broth were tested for degradation of pig bristle to designate the most potent α-helix keratinolytic proteases. The results (Fig. 4a) indicated that all the fractions of culture broth could degrade pig bristle to different degrees. However, fractions C15, C20, A11, and A10 were much more active in pig bristle degradation than the other fractions.

According to the protease identification results (Table 3), fractions A11 and A10 had 12 and 15 proteases, respectively. However, fraction C15 only had three proteases 6877 (S8), 6423 (accession number: KP290880, M28), and 8393 (accession number: KP290873, M3). Fraction C20 also only had four proteases 6877 (S8), 11652 (accession number: KP290866, S8), 6423 (M28), and 8025 (accession number: KP290838, M28). These two fractions (C15 and C20) shared the 6423 and 6877 proteases. The degrading capabilities of fraction C15 and C20 were comparably high or slightly higher than the positive controls with full composition proteases, indicating that the proteases in fraction C15 and C20 play an important role in keratin degradation.

**Fig. 5:**
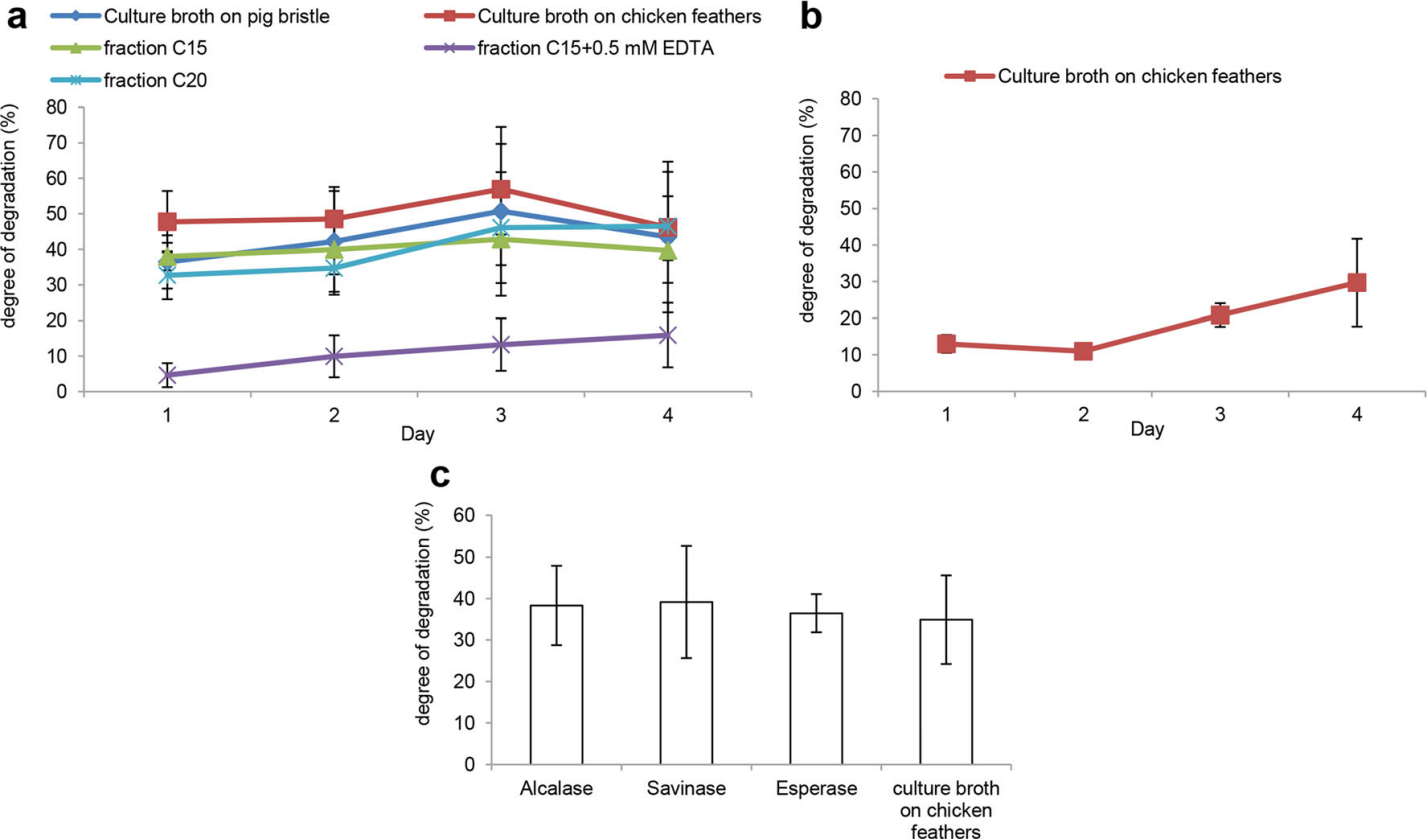
Degradation of keratinaceous materials in a long-term incubation. **a** Pig bristle treated for 4 days with culture broth supernatant and with fractions of culture broth. Culture broth on pig bristle: culture broth supernatant from *O. corvina* grown for 11 days on fermentation medium with pig bristle; culture broth on chichen feathers: culture broth supernatant from *O. corvina* grown for 11 days on fermentation medium with chicken feathers. **b** Bristles and hooves (ground into particles approx. 1–2 mm in diameter) treated for 4 days with culture broth supernatant of *O. corvina* grown on chicken feathers. Negative control: 2× McIlvaine buffer (pH 8). **c** Pig bristle treated for 24 h with culture broth on chicken feathers and commercial keratinolytic proteases (alcalase, savinase, and esperase). The dosage of enzymes was based on the same protein concentration of the keratinolytic proteases in culture broth and the commercial enzymes. The degree of degradation was calibrated to negative control

In a further test of the ability of the proteases to degrade pig bristle, the different fractions C15, C20, and the culture broth from *O. corvina* grown on chicken feathers or pig bristle were incubated with pig bristle at 40 °C and degradation was followed over 4 days. After 3-day incubation, culture broth on chicken feathers, culture broth on pig bristle, fraction C20 and C15 degraded 57, 51, 46, and 43 % of the pig bristle, respectively (Fig. 5a). Moreover, as protease 8393 is an M3 family metalloprotease, the function of this protease in the C15 fraction was evaluated by adding 0.5 mM of the metalloprotease inhibitor EDTA to the C15 fraction. The results showed that addition of EDTA was associated with a strongly decreased degradation of pig bristle (Fig. 5a). Therefore, metalloprotease 8393 (M3) is important for keratin degradation in combination with endopeptidase 6877 (S8) and exopeptidase 6423 (M28) in fraction C15.

To assess the capability of the *O. corvina* proteases for degradation of an industrially relevant substrate, pretreated bristles and hooves obtained from a slaughterhouse were incubated with culture broth from chicken feathers. After 4 days of incubation, the culture broth had degraded 40 % of the pretreated bristles and hooves (Fig. 5b).

The degradation capability was also compared among the commercial keratinolytic proteases (esperase, alcalase, and savinase) and the culture broth on chicken feathers (Fig. 5c). The results indicated that the ability of keratin degradation of *O. corvina* culture broth was very close to the commercial enzymes.

#### Pig bristle degraded by blends composed of most active fractions of culture broth and of the best performing recombinant protease and fractions of culture broth

Synergistic action between the different amounts and types of proteases present in the fractions of culture broth was investigated by mixing the fractions in different combinations and testing for degradation of pig bristle. Enzyme blend produced by mixing of fraction C15 and C20 gave the highest activity (18 %), which was higher than testing the two fractions individually (14 % for each) when adjusted for equal enzyme load (Fig. 4b). Therefore, the enzymes found in C15 and C20 fractions (endoactive protease (two S8), exoactive proteases (two M28), and a metalloprotease (M3)) may have synergistic effect.

Next, we tested the synergistic action between the proteases present in the fractions of culture broth and the purified recombinant protease 6877 (S8). The results indicated that the degree of degradation by the recombinant protease 6877 was 16 %. The blends with recombinant protease 6877 did not show an increased degree of degradation (Fig. 4b). Thus, recombinant protease 6877 has high keratinolytic activity and shares the same substrate specificity as the proteases in the fractions.

## Discussion

A single kind of keratinolytic protease is not sufficient for efficient keratin degradation, and this suggests that degradation of this recalcitrant material requires the cooperative action of multiple enzymes (Yamamura et al. 2002). *O. corvina* can utilize and decompose compact hard keratinaceous materials such as chicken feathers, duck feathers, dog wool, and pig bristle as sole carbon and nitrogen source. Hence, it can be inferred that *O. corvina* is in all probability an effective producer of multiple proteases for keratin decomposition. In this study, we applied the new technology PPR for protease gene mining in the *O. corvina* genome (Busk and Lange 2013). The amino acid sequences of most of the 73 predicted proteases share less than 85 % identity with proteases from dermatophytic ascomyceteous fungi *Arthroderma* sp. and *Trichophyton* sp. that grow on different keratinaceous materials than *O. corvina*.

Comparative analysis of proteases of keratin degraders and non-keratin degraders showed that the most abundant proteases in genomes of keratin degraders were from M36, M35, M43, and S8 families. It was reported that pathogenic fungi mainly secrete endoproteases, including the aspartic proteases of the pepsin family (A1 family), serine proteases of the subtilisin subfamily (S8A), and metalloproteases of two different families (M36 and M35 families) (Monod et al. 2002). Subtilisin-like serine proteases (S8) play an important role in disrupting the mechanical integrity of the keratinaceous substrate (Huang et al. 2004; Li et al. 2010; Walton 1996). The 10 subtilisin-like serine protease genes in the *O. corvina* genome are diverse in protein sequences and belong to different subgroups (Fig. S1). This diversity could suggest that the secreted keratinolytic proteases have evolved over a long time (Hu and Leger 2004; Muszewska et al. 2011). In addition, SUB3 and SUB4 proteases in S8 family are endopeptidases that play an important role in degradation of keratinaceous tissues (Jousson et al. 2004b; Muszewska et al. 2011). So, these two subgroup proteases may also be important for keratin degradation for *O. corvina*. Members of M35 (deuterolysins) and M36 (fungalysins) metalloendopeptidase families have been shown to be involved in degrading keratinaceous substrate (Jousson et al. 2004a; Li et al. 2012; Tarabees et al. 2013). These enzymes can overcome the limited proteolysis on the surface of insoluble keratin particles which restricts enzyme-substrate interaction. The M43 proteases have been identified in dermytophytic genomes (Martinez et al. 2012), but a keratinolytic function has not been described so far.

Only 12 of 18 genes encoding candidate keratinolytic proteases could be amplified from the cDNA when *O. corvina* was grown on keratinaceous substrates. Of the 12 candidate proteases, eight of 10 subtilisin-like serine proteases were expressed at transcriptomic level. However, only recombinant protease 6877 (S8: SUB3) exhibited high keratin-degrading activity. No or low activity of other proteases may be explained by incorrect folding or processing. Propeptide-sequence (Pro-sequence) engineering involving site-directed mutagenesis, truncating the pro-sequence or swapping pro-sequence, were successfully used to produce recombinant keratinolytic proteases (Li et al. 2013; Rajput et al. 2012; Sharma et al. 2011).

Keratinaceous materials are not composed only of keratin but also of an insoluble network of different cross-linked proteins (Simon and Green 1985; Steinert and Marekov 1995). Thus, additional proteases to attack this structure are necessary for decomposition of keratin. We discovered a wide variety of secreted proteases from *O. corvina.* The proteases were mainly metallo and serine proteases, and more M28 and S8 family members were found than of the other proteases. This is a different protease composition than found in *T. rubrum* grown on skin and nails: Metalloprotease (M36: MEP2) and serine proteases (S8: SUB5, SUB2, and SUB3) were more active in skin medium, and serine proteases (S8: SUB1 and SUB4) and metalloprotease (M36: MEP4) were more active in nail degradation (Chen et al. 2010). *O. corvina* protease secretion is also different from *A. benhamiae* which mainly secretes proteases such as serine proteases (S8: SUB3, SUB4, SUB7), metalloprotease (M36: MEP1, MEP3, and MEP4), leucine aminopeptidases LAP1, LAP2, and dipeptidyl peptidases DPPIV and DPPV when it degrades keratin (Burmester et al. 2011). These differences in secretomes between *O. corvina* and dermatophytes may be attributed to the different lineages or the different species that are specialized and adapted to grow on various keratinaceous substrates. However, both endoproteases and exoproteases were detected in all the secretomes, which indicates that these two kinds of enzymes are both expressed at the same time and are possibly involved in keratin degradation.

The presence of protease in the secretome does not necessarily mean that these proteases are actually involved in keratin decomposition. Therefore, we fractionated the culture broth supernatant to pinpoint specific and sufficiently effective keratinolytic activities which we followed by UV absorbance measurements. In our case, soluble protein quantification in pig bristle degradation by UV absorbance is more suitable than by Bradford and BCA methods (Fig. S4). This analysis identified four fractions that showed a high degree of degradation with pig bristle substrate. Interestingly, fraction C15 and C20 only had three and four proteases, respectively, from the S8, M28, and M3 family. The endopeptidase 6877 (S8) combined with the exopeptidase (M28) and metallopeptidase (M3) found in the partially purified fraction C15 showed a much higher degree of pig bristle degradation than that of the single purified recombinant 6877 (S8) protease. Furthermore, two endopeptidases (S8) and two exopeptidases (M28) found in the partially purified fraction C20 also achieved a similar high degree of degradation. Enzyme blend mixing of fraction C15 and C20 gave higher activity than testing the two fractions individually. This result indicates that the endoactive proteases (S8), exoactive proteases (M28), and the metalloprotease (M3), which were able to cleave from the end or middle of the protein structure, could have highly synergistic function for keratinaceous material decomposition. In nature, keratin-degrading fungi are known to secrete sulfite to destabilize the keratin by breaking the cysteine bridges, thereby making the keratin more susceptible to proteolysis and increasing the activity of keratinases threefold (Grumbt et al. 2013; Kunert 1992). Therefore, it is surprising that approx. 50 % degradation of pig bristle can be achieved in a few days with the partially purified proteases in fractions C15 and C20 without addition of sulfite to break the cysteine bridges of the keratin. Even higher levels of degradation might have been achieved by adding sulfite, as reported by Kunert (1992). It is possible that the slightly higher degradation (approx. 60 %) obtained using culture broth was due to the presence of sulfite secreted by *O. corvina* during growth. The culture broth had degraded 30 % of the pretreated bristles and hooves obtained from a slaughterhouse. Meanwhile, the similar degree of degradation among commercial keratinolytic proteases and the culture broth of *O. corvina* further indicate that the proteases secreted by *O. corvina* are able to degrade this industrial waste substrate to a large degree even without addition of sulfite.

The above results suggest that the high keratin-degrading activity observed from culture broth of *O. corvina* originates from five genes belonging to three protein families, and most of such activity can be achieved by using enzyme blends including three to four of these proteases or by only using the protease 6877 (S8). These five genes, which have specific and high α- and β-keratin-degrading capabilities, belong to the same protein families as the well-known keratin-acting proteases from the human pathogens *Trichophyton* sp. Amino acid sequence identity, however, was only between 72 and 84 %.

This sequence difference provides a basis for understanding the improved performance and provides room for further evolution, engineering, mutation, or shuffling of keratinolytic proteases of *Onygena* for even better performance and stability. Meanwhile, this study provides basic information for further investigation of optimized keratin degradation through purification of each protease and making effective enzyme blends from non-pathogenic fungi. Therefore, the five novel genes may have, or can be engineered to have, sufficient activity for decomposition of keratin to bioaccessible proteins, peptides, and amino acids. Such a development could unlock the potential of a very substantial protein resource for use as animal feed. The discovered keratinolytic proteases in the culture broth with broad pH range (Lange et al. 2014) could be also used in leather and fertilizer industries, in production of biohydrogen, for silver recovery from X-ray film and also as detergent additives.

In conclusion, we discovered five novel proteases (6877, 11652, 6423, 8025, and 8393) involved in keratin decomposition from non-pathogenic *O. corvina* using three different approaches: (1) recombinant expression and characterization of putative proteases; (2) analysis of secreted proteases; and (3) fractionation of culture broth supernatant. A blend of three of these five proteases from S8, M28, and M3 families were found to efficiently degrade pig bristle. This result points to that a combination of different exo- and endo-acting proteases can efficiently degrade keratin. The discovery also suggests a basis for developing an industrially relevant enzyme composition to be used to decompose both α- and β-keratin to bioaccessible proteins, peptides, and amino acids, which are a substantial protein resource for use as animal feed.

## Electronic supplementary material

Below is the link to the electronic supplementary material.

ESM 1 (PDF 1313 kb)
