# Supplementary material for: Genome and secretome analyses provide insights into keratin decomposition by novel proteases from the non-pathogenic fungus Onygena corvina
Source: Appl Microbiol Biotechnol. 2015 Jul 16;99(22):9635–49. doi: 10.1007/s00253-015-6805-9 (PMC4628079; doi:10.1007/s00253-015-6805-9)
Supplement: Supplementary file 1 — (PDF 1313 kb) [file 253_2015_6805_MOESM1_ESM.pdf]

**Supplementary material**

# Genome and secretome analyses provide insights into keratin decomposition by novel proteases from the non-pathogenic fungus *Onygena corvina*

**Applied Microbiology and Biotechnology**

**Yuhong Huang · Peter Kamp Busk · Florian-Alexander Herbst · Lene Lange**

Y. Huang · P. K. Busk · L. Lange (Corresponding author)
Department of Chemistry and Bioscience, Aalborg University Copenhagen, 2450 Copenhagen SV, Denmark
New address: Department of Chemical and Biochemical Engineering, Technical University of Denmark, Building 229, 2800 Kgs. Lyngby, Denmark
Phone: +45 24432040; Email: lenl@kt.dtu.dk

F. Herbst
Center for Microbial Communities, Department of Chemistry and Bioscience, Aalborg University, Fredrik Bajers Vej 7H, 9220 Aalborg East, Denmark

## Supplemental tables

**Table S1** Primer sequences for PCR. Restriction site was underlined in each primer sequence. Stop code “TTA” was italicized in each reverse primer. His-tag was added in each reverse primer (bold sequence)

| Gene | Family | Coding gene length (bp) | Anneal-ing Tempe-rature (°C) | Primer | Sequence 5'→3' | Restriction site |
|---|---|---|---|---|---|---|
| 7508 | S8/ SUB1 | 1473 | 55 | Forwad | AAAAGGCCTGGTGTTTTGAAGTTCATTTCCAT | *Stu*I |
| | | | | Reverse | TGCGGCCGGCC*TTA***ATGATGATGATGATGATG**ATGGTGAGACCACCACCAAG | *Fse*I |
| 6266 | S8/ SUB2 | 1248 | 60 | Forwad | TCGAGGATATCCAGCTCTTCAGCCTCCTCCT | *Eco*RV |
| | | | | Reverse | TGCGGCCGGCC*TTA***ATGATGATGATGATGATG**CTGCTGCACCTTGGAGCG | *Fse*I |
| 6877 | S8/ SUB3 | 1182 | 62 | Forwad | AAAAGGCCTGGCTGCATCAAGGTCATCTCCG | *Stu*I |
| | | | | Reverse | TGCGGCCGGCC*TTA***ATGATGATGATGATGATG**TTGGCCGCTTCCGTTGTAGAG | *Fse*I |
| 8702 | S8/ SUB3 | 1197 | 55 | Forwad | AAAAGGCCTTTCTCCCTCAAGACTTTGTCCA | *Stu*I |
| | | | | Reverse | TGCGGCCGGCC*TTA***ATGATGATGATGATGATG**GTATCCGCTTCCGTTGTACA | *Fse*I |
| 8545 | S8/ SUB7 | 1203 | 55 | Forwad | AAAAGGCCTGGTTTCATTACCAAGGCCCTC | *Stu*I |
| | | | | Reverse | TGCGGCCGGCC*TTA***ATGATGATGATGATGATG**CATGCCAGAGCCGTTGTTGAT | *Fse*I |
| 11652 | S8/ SUB7 | 1209 | 60 | Forwad | AAAAGGCCTAAGATCGCGGAGAGAGCATTC | *Stu*I |
| | | | | Reverse | TGCGGCCGGCC*TTA***ATGATGATGATGATGATG**GCGTCCGCTCCCATTGTA | *Fse*I |
| 11813 | S8/ SUB8 | 1473 | 60 | Forwad | AAAAGGCCTAAGGGCCTCCTCAGCCTCTC | *Stu*I |
| | | | | Reverse | TGCGGCCGGCC*TTA***ATGATGATGATGATGATG**TACAATGATGGCGTCCTTGAGCT | *Fse*I |
| 7122 | S8/ SUB9 | 1209 | 58 | Forwad | AAAAGGCCTGGTTTCTTCCGCGCCTTATT | *Stu*I |
| | | | | Reverse | TGCGGCCGGCC*TTA***ATGATGATGATGATGATG**CGCCCCGCTACCGTTGT | *Fse*I |
| 14354 | S8/ SUB11 | 1200 | 58 | Forwad | TCGAGGATATCGGTTTCCTCAAGACCATTGC | *Eco*RV |
| | | | | Reverse | TGCGGCCGGCC*TTA***ATGATGATGATGATGATG**CTGACCGCTACCGTTATACA | *Fse*I |
| 6582 | S8/SU B12 | 1212 | 55 | Forwad | TCGAGGATATCTTTCTCTTGAAACCCTTGGTG | *Eco*RV |
| | | | | Reverse | TGCGGCCGGCC*TTA***ATGATGATGATGATGATG**AGCTCCACTATCGTTGTAGAG | *Fse*I |
| 8301 | M35/ NPIIB | 1101 | 60 | Forwad | TCGAGGATATCCAAGTCATTGTTGCTCTTGCT | *Eco*RV |
| | | | | Reverse | TGCGGCCGGCC*TTA***ATGATGATGATGATGATG**GCAGCCAACGTAGATAGCG | *Fse*I |
| 7758 | M35/ NPIID | 1098 | 60 | Forwad | TCGAGGATATCCAATTTATTGCTGCCCTCTCG | *EcoR*V |
| | | | | Reverse | TGCGGCCGGCC*TTA***ATGATGATGATGATGATG**GCAGTTGACATAGATGGCATT | *Fse*I |
| 11002 | M36/ MEP1 | 1899 | 60 | Forwad | AAAAGGCCTCACGGTTTACTCCTTGCCG | *Stu*I |
| | | | | Reverse | TGCGGCCGGCC*TTA***ATGATGATGATGATGATG**CTGCACTCTGGTGGGACA | *Fse*I |
| 12526 | M36/ MEP2 | 1884 | 60 | Forwad | AAAAGGCCTCACGGACTCTTGCTCGCCG | *Stu*I |
| | | | | Reverse | TGCGGCCGGCC*TTA***ATGATGATGATGATGATG**GCAAGCACCGTCGAAAGACATG | *Fse*I |
| 8814 | M36/ MEP3 | 1902 | 62 | Forwad | TCGAGGATATCCACGGCCTCTTGCTTGCC | *Eco*RV |
| | | | | Reverse | TGCGGCCGGCC*TTA***ATGATGATGATGATGATG**GCATCCTGGGGGGAGCTG | *Fse*I |
| 3998 | M36/ MEP4 | 1905 | 60 | Forwad | AAAAGTACTCACGGCCTTCTGCTTGCTG | *Sca*I |
| | | | | Reverse | TGCGGCCGGCC*TTA***ATGATGATGATGATGATG**ACATCCTTCAGGGAGCTTGTT | *Fse*I |
| 3705 | M43/ MEP6 | 813 | 60 | Forwad | AAAAGGCCTCGTCTCTCTGTCCTTCTCTCCG | *Stu*I |
| | | | | Reverse | TGCGGCCGGCC*TTA***ATGATGATGATGATGATG**GTATGGAGCACGGAACGCAT | *Fse*I |
| 6296 | M43/ MEP8 | 822 | 60 | Forwad | AAAAGGCCTCGTGTCTCTCTATCTCTTCTTGGC | *Stu*I |
| | | | | Reverse | TGCGGCCGGCC*TTA***ATGATGATGATGATGATG**TTTGCCGGCACGCAGCT | *Fse*I |

**Table S2** Prediction results of protease genes in *O. corvina* genome by PPR. The nucleotide sequences have been deposited at GenBank under accession numbers KP290810-KP290882

| Name | Clan | Family | Description of the best blast hit | Identity (%) | Accession |
|---|---|---|---|---|---|
| 10067 | CF | C15 | pyroglutamyl peptidase type I, putative [*Arthroderma benhamiae* CBS 112371] | 64 | KP290844 |
| 5296 | PC | C56 | DJ-1/PfpI family protein [*Arthroderma otae* CBS 113480] | 86 | KP290845 |
| 11832 | PC | C56 | DJ-1/PfpI family protein [*Arthroderma gypseum* CBS 118893] | 71 | KP290846 |
| 13473 | PC | C56 | ThiJ/PfpI family protein [*Arthroderma benhamiae* CBS 112371] | 82 | KP290847 |
| 5775 | MA | M1 | aminopeptidase 2 [*Arthroderma otae* CBS 113480] | 87 | KP290872 |
| 8393 | MA | M3 | thimet oligopeptidase [*Arthroderma otae* CBS 113480] | 90 | KP290873 |
| 1987 | MA | M12 | zinc metalloprotease mde10 [*Arthroderma gypseum* CBS 118893] | 97 | KP290848 |
| 7488 | MC | M14 | zinc carboxypeptidase [*Trichophyton tonsurans* CBS 112818] | 93 | KP290849 |
| 11012 | MC | M14 | carboxypeptidase 2 [*Arthroderma otae* CBS 113480] | 81 | KP290850 |
| 2945 | MC | M14 | RecName: Full=Metallocarboxypeptidase A [*Trichophyton equinum*] | 88 | KP290851 |
| 7142 | MC | M14 | carboxypeptidase A4 [*Arthroderma otae* CBS 113480] | 80 | KP290852 |
| 2112 | MH | M20 | glutamate carboxypeptidase [*Arthroderma otae* CBS 113480] | 89 | KP290853 |
| 3755 | MH | M20 | peptidase family M20/M25/M40 protein [*Ajellomyces dermatitidis* ATCC 18188] | 62 | KP290854 |
| 8832 | MH | M20 | peptidase, putative [*Arthroderma benhamiae* CBS 112371] | 79 | KP290855 |
| 2014 | MH | M20 | N-carbamoyl-L-amino acid hydrolase [*Arthroderma otae* CBS 113480] | 93 | KP290856 |
| 8301 | MA | M35 | neutral protease 2 [*Arthroderma gypseum* CBS 118893] | 77 | KP290810 |
| 7758 | MA | M35 | metalloproteinase [*Arthroderma gypseum* CBS 118893] | 84 | KP290811 |
| 12526 | MA | M36 | extracellular elastinolytic metalloproteinase [*Arthroderma otae* CBS 113480] | 85 | KP290812 |
| 8814 | MA | M36 | metalloprotease Mep3 [*Trichophyton equinum*] | 89 | KP290813 |
| 3998 | MA | M36 | metalloprotease Mep4 [*Trichophyton tonsurans*] | 80 | KP290814 |
| 11002 | MA | M36 | putative secreted metalloprotease 1, partial [*Arthroderma uncinatum*] | 89 | KP290815 |
| 3705 | MA | M43 | metalloprotease 1 [*Arthroderma gypseum* CBS 118893] | 95 | KP290816 |
| 6296 | MA | M43 | metalloprotease 1 [*Arthroderma gypseum* CBS 118893] | 75 | KP290817 |
| 1764 | MA | M43 | metalloprotease 1 [*Arthroderma otae* CBS 113480] | 80 | KP290857 |
| 13394 | MA | M49 | dipeptidyl-peptidase 3 [*Arthroderma otae* CBS 113480] | 91 | KP290874 |
| 991 | SB | S8 | kex protein [*Arthroderma otae* CBS 113480] | 79 | KP290869 |
| 6266 | SB | S8 | Subtilisin-like protease 2 [*Arthroderma benhamiae* CBS 112371] | 77 | KP290870 |
| 14286 | SB | S8 | serine peptidase [*Arthroderma gypseum* CBS 118893] | 78 | KP290871 |
| 6582 | SB | S8 | alkaline serine protease [*Trichophyton tonsurans* CBS 112818] | 64 | KP290859 |
| 6877 | SB | S8 | alkaline serine protease [*Trichophyton tonsurans* CBS 112818] | 84 | KP290860 |
| 7122 | SB | S8 | alkaline proteinase [*Arthroderma otae* CBS 113480] | 71 | KP290861 |
| 7508 | SB | S8 | Subtilisin-like protease 1 [*Arthroderma benhamiae* CBS 112371] | 72 | KP290862 |
| 8096 | SB | S8 | subtilisin, putative [*Metarhizium anisopliae* ARSEF 23] | 39 | KP290863 |
| 8545 | SB | S8 | Subtilisin-like protease 7 [*Trichophyton interdigitale* H6] | 81 | KP290864 |
| 8702 | SB | S8 | Subtilisin-like protease 4 [*Trichophyton tonsurans*] | 85 | KP290865 |
| 11652 | SB | S8 | alkaline serine protease [*Trichophyton equinum* CBS 127.97] | 70 | KP290866 |
| 11813 | SB | S8 | serine proteinase [*Arthroderma otae* CBS 113480] | 90 | KP290867 |

| | | | | | |
|---|---|---|---|---|---|
| 14354 | SB | S8 | alkaline serine protease [*Trichophyton rubrum* CBS 118892] | 64 | KP290868 |
| 9005 | SC | S9 | dipeptidyl peptidase 4 [*Arthroderma otae* CBS 113480] | 87 | KP290875 |
| 346 | SC | S9 | dipeptidyl-peptidase 5 [*Arthroderma otae* CBS 113480] | 87 | KP290876 |
| 4005 | SC | S10 | carboxypeptidase Y [*Arthroderma otae* CBS 113480] | 73 | KP290877 |
| 12542 | SC | S10 | serine carboxypeptidase [*Trichophyton rubrum* CBS 118892] | 79 | KP290878 |
| 10291 | SC | S28 | serine peptidase, family S28 [*Trichophyton rubrum* CBS 118892] | 85 | KP290818 |
| 12996 | SC | S28 | extracelular serine carboxypeptidase [*Trichophyton equinum* CBS 127.97] | 89 | KP290819 |
| 8582 | SC | S33 | hydrolase [*Arthroderma otae* CBS 113480] | 83 | KP290820 |
| 2416 | SC | S33 | abhydrolase domain-containing protein 9 [*Arthroderma otae* CBS 113480] | 79 | KP290821 |
| 5586 | SC | S33 | proline iminopeptidase [*Arthroderma otae* CBS 113480] | 74 | KP290822 |
| 5164 | SC | S33 | phosphatase methylesterase [*Trichophyton equinum* CBS 127.97] | 67 | KP290823 |
| 4194 | SC | S33 | epoxide hydrolase [*Arthroderma otae* CBS 113480] | 82 | KP290824 |
| 3985 | SC | S33 | proline iminopeptidase [*Arthroderma otae* CBS 113480] | 87 | KP290825 |
| 12641 | SC | S33 | abhydrolase domain-containing protein [*Arthroderma gypseum* CBS 118893] | 76 | KP290826 |
| 4828 | SC | S33 | epoxide hydrolase [*Trichophyton equinum* CBS 127.97] | 79 | KP290827 |
| 14115 | SC | S33 | abhydrolase domain-containing protein 4 [*Arthroderma gypseum* CBS 118893] | 79 | KP290828 |
| 11576 | MA | S41 | peptidase S41 family protein [*Arthroderma gypseum* CBS 118893] | 78 | KP290879 |
| 4515 | SB | S53 | serine protease, putative [*Arthroderma benhamiae* CBS 112371] | 89 | KP290829 |
| 1027 | SB | S53 | tripeptidyl peptidase SED3 [*Arthroderma otae* CBS 113480] | 80 | KP290830 |
| 12481 | SB | S53 | aspartic-type endopeptidase (OpsB), putative [*Arthroderma benhamiae* CBS 112371] | 82 | KP290831 |
| 1617 | MRG | | keratinolytic protein [*Trichophyton rubrum*] | 88 | KP290858 |
| 3474 | MH | M18 | aspartyl aminopeptidase [*Arthroderma otae* CBS 113480] | 81 | KP290832 |
| 6343 | MH | M18 | aspartyl aminopeptidase [*Arthroderma otae* CBS 113480] | 94 | KP290833 |
| 5086 | MJ | M19 | membrane dipeptidase GliJ [*Arthroderma otae* CBS 113480] | 81 | KP290834 |
| 12364 | MJ | M19 | dipeptidase 1 [*Arthroderma otae* CBS 113480] | 92 | KP290835 |
| 4132 | MH | M28 | glutamate carboxypeptidase [*Trichophyton equinum* CBS 127.97] | 78 | KP290837 |
| 8025 | MH | M28 | leucyl aminopeptidase [*Arthroderma otae* CBS 113480] | 81 | KP290838 |
| 13646 | MH | M28 | glutamate carboxypeptidase 2 [*Arthroderma otae* CBS 113480] | 79 | KP290839 |
| 7705 | MH | M28 | leucyl aminopeptidase [*Arthroderma otae* CBS 113480] | 69 | KP290840 |
| 645 | MH | M28 | leucyl aminopeptidase [*Arthroderma otae* CBS 113480] | 76 | KP290841 |
| 10892 | MH | M28 | glutamate carboxypeptidase [*Trichophyton tonsurans* CBS 112818] | 90 | KP290842 |
| 6844 | MH | M28 | peptidase M28 [*Arthroderma otae* CBS 113480] | 90 | KP290843 |
| 6423 | MH | M28 | leucine aminopeptidase 1 [*Arthroderma otae* CBS 113480] | 83 | KP290880 |
| 3032 | MH | M28 | peptidase [*Trichophyton tonsurans* CBS 112818] | 76 | KP290836 |
| 8472 | - | - | alanine-glyoxylate aminotransferase [*Paracoccidioides* sp.] | 75 | KP290881 |
| 13395 | - | - | ornithine aminotransferase [*Arthroderma otae* CBS 113480] | 86 | KP290882 |

**Table S3** Assembly quality of *O. corvina* genome

| | Length[a] | Length[b] |
|---|---|---|
| $N_{75}$ | 160383 | 125887 |
| $N_{50}$ | 260639 | 224872 |
| $N_{25}$ | 517260 | 324265 |
| Minimum | 198 | 120 |
| Maximum | 933412 | 922970 |
| Average | 22096 | 18022 |
| Count | 992 | 1216 |
| Total | 21919116 | 21914624 |

[a] Contig measurements (including scaffolded regions);

[b] Contig measurements (excluding scaffolded regions)

**Table S4** Summary statistics of *O. corvina* genome assembly

| | Count | Average length | Total bases |
|---|---|---|---|
| Reads | 81538322 | 78.47 | 6398237900 |
| Matched | 80660745 | 78.65 | 6343750062 |
| Not matched | 877577 | 62.09 | 54487838 |
| Contigs | 992 | 22096 | 21919116 |
| Reads in pairs | 74474370 | 182.91 | |
| Broken paired reads | 6186375 | 76.37 | |

**Table S5** Proteases found in secretome of *O. corvina* growing on chicken feathers (C) or pig bristle (P), n=3

| Predicted gene by PPR | Fami-ly | Annotation | Peptides-C1 | Peptides-C2 | Peptides-C3 | Peptides-P1 | Peptides-P2 | Peptides-P3 | $log_2$ C/P | (* <0.05. **<0.01) | *p*-value | LFQ C | LFQ P |
|---|---|---|---|---|---|---|---|---|---|---|---|---|---|
| 6877 | S8 | Subtilisin-like protease 3 | 6 | 8 | 7 | 7 | 7 | 7 | 2.82 | * | 0.02 | 4.206E+10 | 5.943E+09 |
| 6877 | S8 | Subtilisin-like protease 3 | 14 | 15 | 15 | 15 | 16 | 16 | 2.66 | * | 0.04 | 6.323E+10 | 1.001E+10 |
| 8393 | M3 | metallopeptidase | 17 | 17 | 17 | 14 | 16 | 11 | 2.24 | * | 0.05 | 444416667 | 94091667 |
| 4005 | S10 | carboxypeptidase Y | 5 | 13 | 8 | 7 | 5 | 6 | 2.12 | | 0.43 | 466052000 | 107268000 |
| 6844 | M28 | peptidase | 18 | 16 | 17 | 17 | 18 | 15 | 1.59 | ** | 0.01 | 3.637E+09 | 1.207E+09 |
| 10291 | S28 | Serine carboxypeptidase | 13 | 15 | 14 | 10 | 11 | 10 | 1.46 | | 0.26 | 1.18E+09 | 429480000 |
| 8393 | M3 | metallopeptidase | 3 | 2 | 3 | 3 | 3 | 3 | 1.40 | | 0.13 | 26320333 | 9957600 |
| 2945 | M14 | metallocarboxypeptidase | 3 | 4 | 4 | 2 | 3 | 3 | 1.30 | * | 0.03 | 190533333 | 77488000 |
| 3998 | M36 | metalloprotease | 4 | 3 | 3 | 3 | 3 | 3 | 1.21 | * | 0.02 | 835523333 | 360386667 |
| 3998 | M36 | metalloprotease | 8 | 10 | 9 | 12 | 11 | 11 | 1.21 | * | 0.05 | 2.638E+09 | 1.141E+09 |
| 3998 | M36 | metalloprotease | 6 | 7 | 7 | 3 | 4 | 2 | 1.04 | | 0.11 | 1.155E+09 | 562423333 |
| 12542 | S10 | carboxypeptidase | 5 | 6 | 4 | 8 | 8 | 8 | 1.01 | | 0.05 | 293086667 | 145426667 |
| 11576 | S49 | peptidase S41 family protein | 1 | 4 | 4 | 3 | 3 | 4 | 0.99 | | 0.26 | 55990000 | 28259333 |
| 6423 | M28 | leucine aminopeptidase 1 | 8 | 7 | 7 | 7 | 9 | 8 | 0.74 | | 0.13 | 3.983E+09 | 2.38E+09 |
| 11012 | M14 | carboxypeptidase 2 | 5 | 5 | 2 | 3 | 4 | 3 | 0.74 | | 0.43 | 50168000 | 30047500 |
| 6844 | M28 | Peptidase | 10 | 11 | 11 | 11 | 10 | 10 | 0.72 | * | 0.03 | 1.672E+09 | 1.018E+09 |
| 14354 | S8 | subtilisin-like protease. putative | 6 | 6 | 5 | 7 | 7 | 6 | 0.70 | | 0.14 | 490780000 | 301300000 |
| 6423 | M28 | leucine aminopeptidase 1 | 20 | 24 | 24 | 21 | 21 | 19 | 0.57 | | 0.36 | 1.001E+10 | 6.751E+09 |
| 10892 | M28 | glutamate carboxypeptidase | 2 | 2 | 2 | 2 | 3 | 2 | 0.40 | | 0.42 | 34197667 | 25905000 |
| 346 | S9 | Dipeptidyl-peptidase 5 | 9 | 9 | 10 | 16 | 18 | 17 | 0.32 | | 0.31 | 164346667 | 131580000 |
| 9005 | S9 | Dipeptidyl peptidase 4 | 37 | 53 | 35 | 45 | 46 | 41 | 0.20 | | 0.64 | 3.242E+09 | 2.817E+09 |
| 13395 | - | ornithine aminotransferase | 9 | 7 | 7 | 7 | 8 | 6 | 0.12 | | 0.81 | 174663333 | 161231000 |
| 6266 | S8 | Subtilisin-like protease 2 | 9 | 10 | 8 | 9 | 10 | 10 | 0.10 | | 0.82 | 1.143E+09 | 1.066E+09 |
| 11652 | S8 | alkaline serine protease | 4 | 3 | 3 | 4 | 4 | 2 | 0.07 | | 0.87 | 75058667 | 71499667 |
| 13395 | - | ornithine aminotransferase | 3 | 3 | 3 | 3 | 3 | 3 | -0.08 | | 0.89 | 108321000 | 114172333 |
| 4132 | M28 | glutamate carboxypeptidase | 2 | 4 | 2 | 5 | 5 | 4 | -0.12 | | 0.59 | 41377333 | 45107667 |
| 11813 | S8 | serine proteinase | 11 | 12 | 13 | 13 | 12 | 11 | -0.20 | | 0.41 | 612603333 | 702036667 |
| 14354 | S8 | subtilisin-like protease. putative | 2 | 3 | 2 | 3 | 2 | 1 | -0.46 | | 0.50 | 118184667 | 162506667 |
| 8832 | M20 | Peptidase | 17 | 18 | 20 | 24 | 26 | 24 | -0.53 | | 0.19 | 431010000 | 623106667 |
| 6423 | M28 | leucine aminopeptidase 1 | 12 | 15 | 12 | 15 | 15 | 13 | -0.86 | | 0.13 | 3.428E+09 | 6.203E+09 |

| | | | | | | | | | | | | | | |
|---|---|---|---|---|---|---|---|---|---|---|---|---|---|---|
| 10992 | M36 | putative secreted metalloprotease 1 | 2 | 3 | 2 | 8 | 8 | 7 | -1.30 | | 0.22 | 61269667 | 150591000 |
| 5775 | M1 | aminopeptidase 2 | 12 | 8 | 14 | 27 | 26 | 23 | -1.45 | * | 0.05 | 120636667 | 330723333 |
| 13394 | M49 | dipeptidyl-peptidase 3 | 14 | 15 | 15 | 19 | 18 | 16 | -1.46 | ** | 0.00 | 368076667 | 1.013E+09 |
| 13394 | M49 | dipeptidyl-peptidase 3 | 15 | 15 | 12 | 23 | 24 | 21 | -1.48 | ** | 0.00 | 379893333 | 1.058E+09 |
| 346 | S9 | Dipeptidyl-peptidase 5 | 3 | 3 | 3 | 3 | 3 | 3 | -1.51 | | 0.11 | 36272667 | 103643333 |
| 3032 | M28 | peptidase | 3 | 5 | 3 | 12 | 11 | 10 | -1.74 | * | 0.04 | 22056333 | 73750333 |
| 6266 | S8 | Subtilisin-like protease 2 | 5 | 3 | 5 | 6 | 4 | 7 | -1.91 | | 0.11 | 134720000 | 505950000 |
| 6343 | M18 | aspartyl aminopeptidase | 6 | 5 | 8 | 15 | 15 | 12 | -1.97 | * | 0.03 | 344616667 | 1.352E+09 |
| 13394 | M49 | dipeptidyl-peptidase 3 | 2 | 2 | 2 | 3 | 3 | 3 | -2.00 | * | 0.03 | 38922000 | 155966667 |
| 3998 | M36 | metalloprotease | 5 | 5 | 5 | 9 | 9 | 11 | -2.11 | | 0.12 | 652180000 | 2.812E+09 |
| 8472 | - | Aspartate aminotransferase | 4 | 4 | 5 | 18 | 18 | 19 | -2.32 | * | 0.04 | 134986000 | 673220000 |
| 8025 | M28 | leucyl aminopeptidase | 2 | 3 | 2 | 10 | 9 | 10 | -3.40 | | 0.24 | 26078667 | 274760000 |
| 7142 | M14 | carboxypeptidase A4 | 2 | 2 | 2 | 5 | 5 | 4 | -3.95 | ** | 0.00 | 13039000 | 201663333 |

**Table S6** Protease activity profile of selected fractions (2.5 ml) from cation exchange chromatography. 50 ml culture supernatant was diluted to 100 ml sample prior to load. Negative control: 50 mM citric acid buffer (pH 3.86)

| Fraction number | Fraction start (min) | Fraction end (min) | Protease activity (U) |
|---|---|---|---|
| 5 | 36.96 | 37.46 | 1.4 |
| 8 | 38.46 | 38.96 | 1.7 |
| 11 | 39.96 | 40.46 | 0.4 |
| 14 | 41.46 | 41.96 | 13.1 |
| 17 | 42.96 | 43.46 | 7.7 |
| 20 | 44.46 | 44.96 | 48.4 |
| 23 | 45.96 | 46.46 | 34.1 |
| 26 | 47.46 | 47.96 | 17.1 |
| 29 | 48.96 | 49.46 | 0.9 |
| 32 | 50.46 | 50.96 | 0.7 |
| 35 | 51.96 | 52.46 | 0.8 |
| 38 | 53.46 | 53.96 | -0.3 |
| 41 | 54.96 | 55.46 | 0.4 |
| 44 | 56.46 | 56.96 | 0.6 |
| 47 | 57.96 | 58.46 | 0.4 |
| 50 | 59.46 | 59.96 | 0.6 |
| 53 | 60.96 | 61.46 | 0.2 |
| 56 | 62.46 | 62.96 | -0.4 |
| 59 | 63.96 | 64.46 | 1.2 |
| Sample prior to loading | - | - | 17 |
| negative control | - | - | -0.1 |

**Table S7** Protease activity profile of selected fractions (0.85 ml) from anion exchange chromatography. Negative control: 20 mM Tris-HCl buffer (pH 8.6)

| Fraction number | Fraction start (min) | Fraction end (min) | Protease activity (U) |
|---|---|---|---|
| 7 | 65.14 | 66.14 | 2.2 |
| 9 | 67.14 | 68.14 | 26.7 |
| 13 | 71.14 | 72.14 | 12.5 |
| 15 | 73.14 | 74.14 | 11.1 |
| 17 | 75.14 | 76.14 | 2.9 |
| 19 | 77.14 | 78.14 | 4.3 |
| 21 | 79.14 | 80.14 | 3.5 |
| 23 | 81.14 | 82.14 | 2.9 |
| 25 | 83.14 | 84.14 | 2.2 |
| 27 | 85.14 | 86.14 | 2.5 |
| 29 | 87.14 | 88.14 | 3.2 |
| 31 | 89.14 | 90.14 | 4.2 |
| 33 | 91.14 | 92.14 | 3.6 |
| 35 | 93.14 | 94.14 | 2.6 |
| 37 | 95.14 | 96.14 | 1.5 |
| 39 | 97.14 | 98.14 | 1.1 |
| 41 | 99.14 | 100.14 | 0.7 |
| 43 | 101.14 | 102.14 | 0.2 |
| 45 | 103.14 | 104.14 | 0.2 |
| Pre flow through | - | - | 49.7 |
| Load flow through | - | - | 55.6 |
| Wash flow through | - | - | 44.9 |
| negative control | - | - | -0.5 |

**Table S8** Protease identification by LC-MS/MS for fractions with protease activity (azocasein as substrate). A: Anion exchanged fractions; C: Cation exchanged fractions

| Predict-ed gene by PPR | Fami-ly | Annotation | A -8 | A -9 | A-10 | A-11 | A-12 | A-13 | A-14 | A-15 | A-16 | A-17 | A-18 | A-19 | A-20 | C-12 | C-13 | C-14 | C-15 | C-16 | C-17 | C-18 | C-19 | C-20 | C-21 | C-22 | C-23 | C-24 | C-25 | C-26 | C-27 | C-28 | C-29 | C-30 |
|---|---|---|---|---|---|---|---|---|---|---|---|---|---|---|---|---|---|---|---|---|---|---|---|---|---|---|---|---|---|---|---|---|---|---|
| 14354 | S8 | serine protease | + | + | + | + | + | + | + | + | - | - | - | - | - | - | - | - | - | - | - | - | - | - | - | - | - | - | + | + | + | - | - | - |
| 5775 | M1 | aminopeptidase 2 | - | - | - | + | - | - | + | + | - | - | - | - | - | - | - | - | - | - | - | - | - | - | - | - | - | - | - | - | - | - | - | - |
| 12542 | S10 | carboxypeptidase | - | - | - | - | - | - | - | - | - | - | - | - | - | + | + | + | - | + | - | - | - | - | - | - | - | - | - | - | - | - | - | - |
| 11652 | S8 | alkaline serine protease | - | - | - | - | - | - | - | - | - | - | - | - | - | - | - | - | - | - | - | + | + | + | + | - | - | - | - | - | - | - | - | - |
| 2945 | M14 | metallocarboxypeptidases | + | + | + | - | + | + | - | - | + | + | + | - | + | - | - | - | - | - | - | - | - | - | - | - | - | - | - | - | - | - | - | - |
| 6423 | M28 | leucine aminopeptidase | + | + | + | + | + | + | + | + | + | + | + | + | + | + | + | + | + | + | + | + | + | + | + | + | + | + | + | + | + | + | + | + |
| 6343 | M18 | aminopeptidase | - | - | - | - | - | - | - | - | - | - | - | - | + | + | - | - | - | - | - | - | - | - | - | - | - | + | - | - | - | + | - | - |
| 8393 | M3 | metallopeptidase | - | + | + | + | + | + | + | + | + | + | + | + | + | - | - | + | + | + | + | + | - | - | - | - | - | - | - | - | - | - | - | - |
| 6844 | M28 | peptidase | + | + | + | + | - | + | + | + | - | - | + | - | - | - | - | - | - | - | - | - | - | - | - | - | - | - | - | - | - | - | - | - |
| 8832 | M20 | peptidase | + | - | + | + | + | + | + | + | - | - | + | + | - | - | - | - | - | - | - | - | - | - | - | - | - | - | - | - | - | - | - | - |
| 10892 | M28 | glutamate carboxypeptidase | - | - | - | - | - | + | + | + | - | - | + | + | + | - | - | - | - | - | - | - | - | - | - | - | - | - | - | - | - | - | - | - |
| 10291 | S28 | Serine carboxypeptidase | + | + | + | - | - | + | + | - | - | - | + | + | - | - | - | - | - | - | - | - | - | - | - | - | - | - | - | - | - | - | - | - |
| 4132 | M28 | glutamate carboxypeptidase | - | - | - | - | - | + | - | - | + | + | + | + | + | - | - | - | - | - | - | - | - | - | - | - | - | - | - | - | - | - | - | - |
| 11813 | S8 | serine proteinase | + | + | + | + | + | + | + | + | + | + | + | + | + | - | - | - | - | - | - | - | - | - | - | - | - | - | - | - | - | - | - | - |
| 8472 | - | Aspartate aminotransferase | + | + | + | + | - | + | + | + | - | - | + | + | - | - | - | - | - | - | - | + | - | - | - | - | - | - | - | - | - | - | - | - |
| 7142 | M14 | carboxypeptidase | - | - | + | + | + | + | + | + | + | + | + | + | - | - | - | - | - | - | - | - | - | - | - | - | - | - | - | - | - | - | - | - |
| 9005 | S9 | Dipeptidyl peptidase 4 | + | + | + | - | - | + | - | - | - | - | - | - | - | - | - | - | - | - | - | - | - | - | - | - | - | - | - | - | - | - | - | - |
| 8025 | M28 | leucyl aminopeptidase | - | - | - | - | - | + | - | - | - | - | - | - | - | - | - | - | - | + | - | - | + | + | + | - | - | + | + | + | + | + | + | + |
| 13394 | M49 | dipeptidyl-peptidase 3 | + | + | + | + | - | + | + | + | + | + | + | + | + | - | - | - | - | - | - | - | - | - | - | - | - | - | - | - | - | - | - | - |
| 6877 | S8 | serine protease | + | + | + | + | + | + | + | + | + | + | + | + | + | + | + | + | + | + | + | + | + | + | + | + | + | + | + | + | + | + | + | + |
| 3998 | M36 | metallopeptidase | + | + | + | + | + | + | + | + | + | + | + | + | + | + | - | + | - | - | + | - | - | - | - | - | - | - | - | + | - | - | - | - |
| 4005 | S10 | carboxypeptidase | + | + | + | - | - | + | - | - | - | - | - | - | - | - | - | - | - | - | - | - | - | - | - | - | - | + | - | - | + | + | + | - |
| 6266 | S8 | serine protease | - | - | - | - | - | - | - | - | - | - | - | - | - | + | + | - | - | - | - | - | - | - | - | - | - | - | - | - | - | - | - | - |
| | Total proteases | | 1-3 | 1-3 | 15 | 12 | 9 | 18 | 14 | 13 | 9 | 9 | 14 | 12 | 10 | 6 | 4 | 5 | 3 | 5 | 4 | 5 | 4 | 4 | 4 | 2 | 2 | 5 | 4 | 5 | 5 | 5 | 4 | 3 |

## Supplemental figures

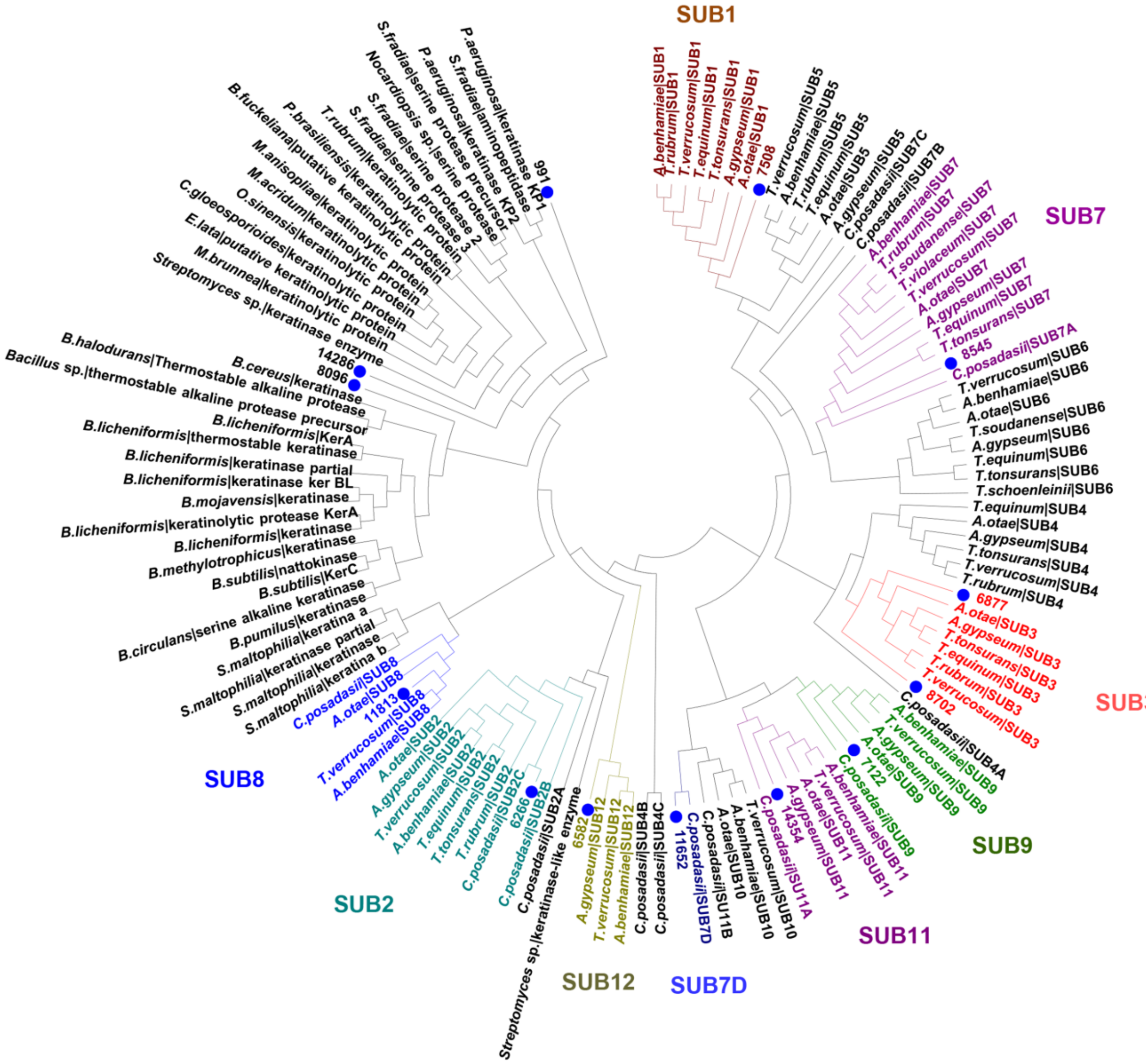


**Fig. S1** Phylogenetic tree of S8 family proteases including those from *O. corvina*. Subtilisin subgroups (SUB) represent functional different proteases, which may have evolved by a series of subtilisin serine protease gene. The colored text indicates the proteases in *O. corvina* genome belonging to different SUB groups

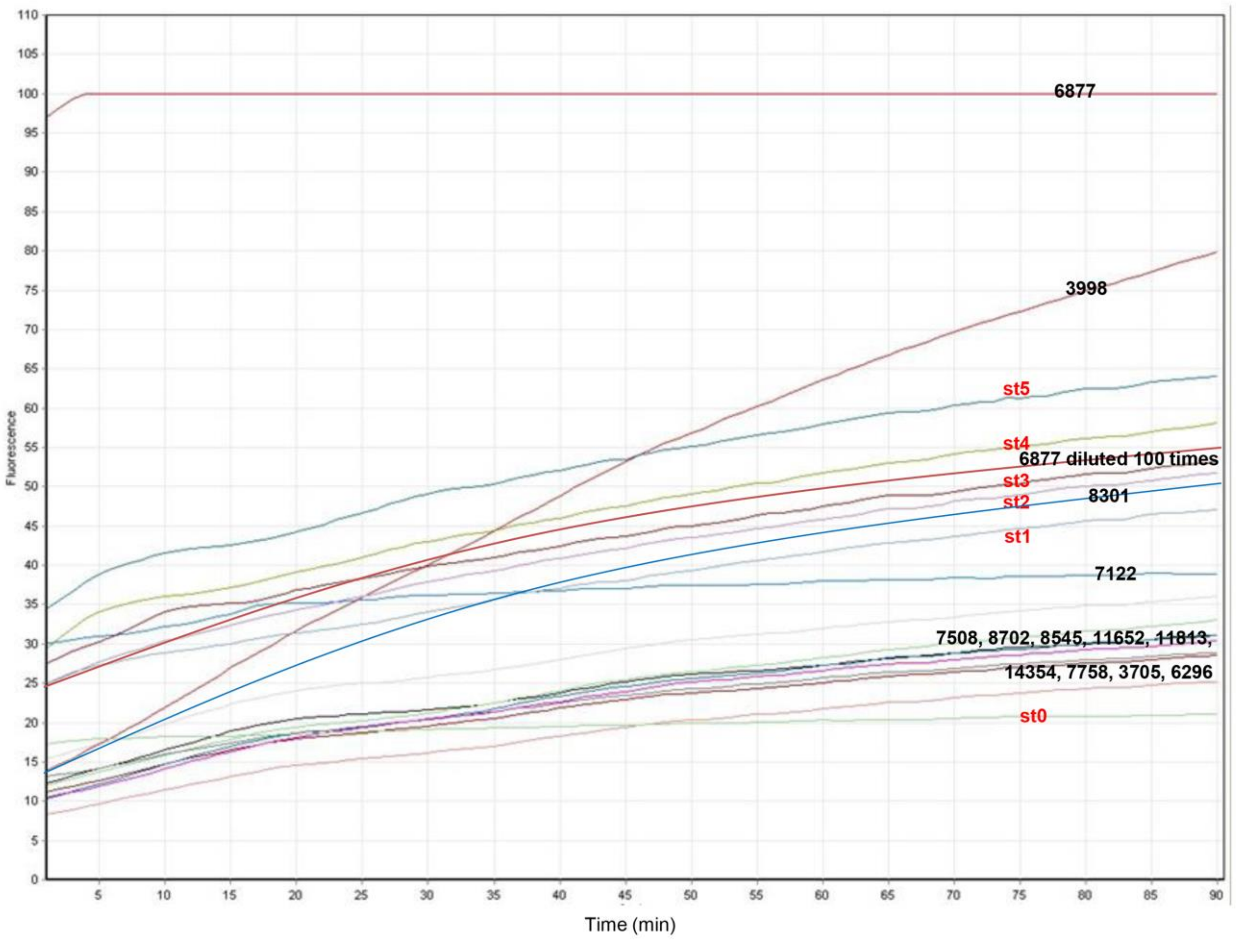


**Fig. S2** Response curves of the fluorescence, indicating level of activity, of each purified recombinant protease. The protease activities are named by the gene. Trypsin standards at the following concentrations: st0 (0 ng/ml trypsin), st1 (100 ng/ml trypsin), st2 (200 ng/ml trypsin), st3 (300 ng/ml trypsin), st4 (400 ng/ml trypsin), st5 (500 ng/ml trypsin)

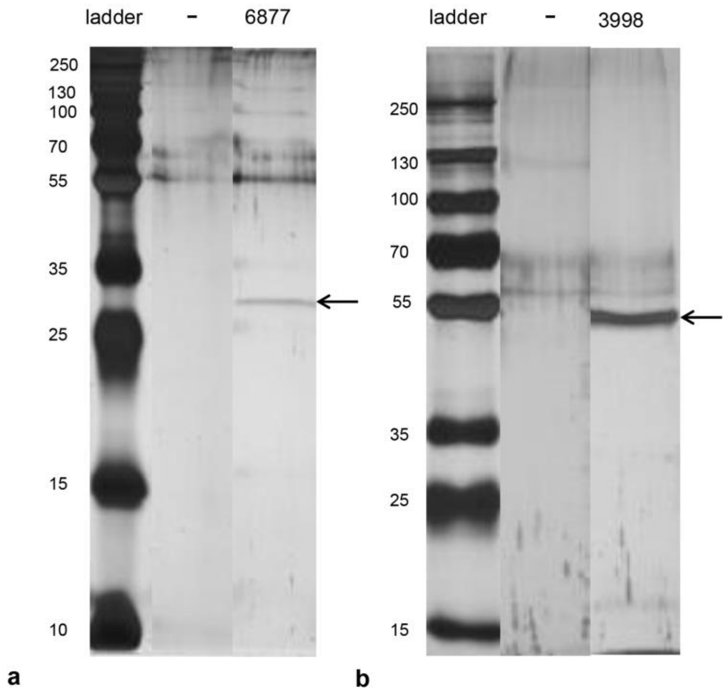


**Fig. S3** SDS-PAGE of purified recombinant proteases. Proteases 6877 (13 µg) and 3998 (8 µg) were loaded on 12 % and 10 % (w/v) polyacrylamide gel, respectively. “-” indicates the purified supernatant of PichiaPink Strain 4 which harbored empty vector pPinkα-HC. Gels were silver stained using the Pierce Silver Stain Kit (Thermo scientific). Ladder: Page Ruler Plus Pre stained Protein Ladder, 10 to 250 kDa

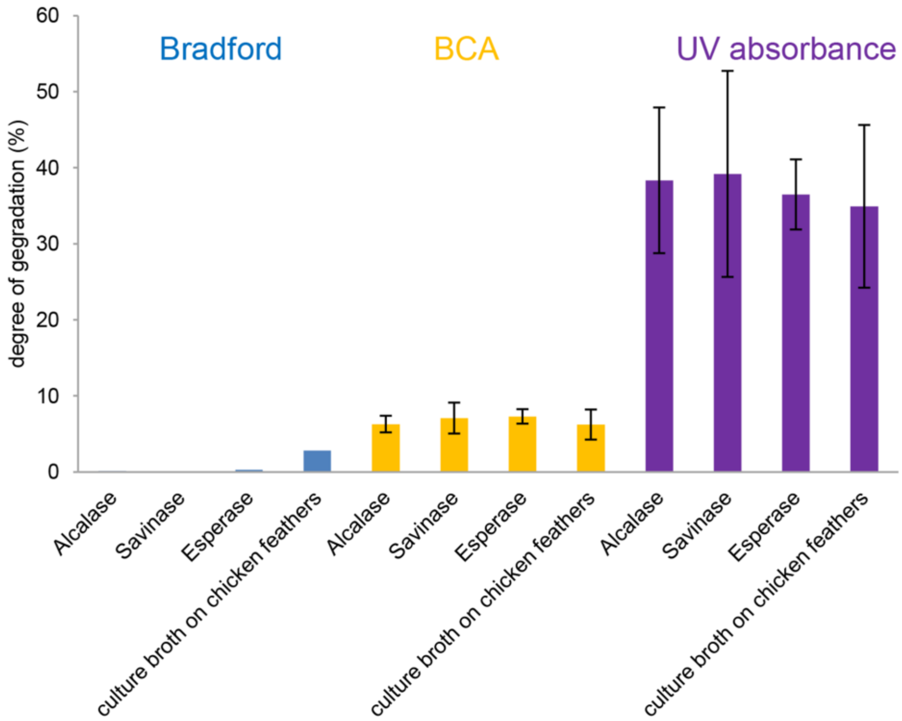


**Fig. S4** Degree of degradation of pig bristle by commercial keratinases (Alcalase, Savinase and Esperase) and culture broth after 24 h of incubation. Soluble protein was measured by Bradford protein assay, BCA protein assay (Pierce BCA Protein Assay Kit, 23225, Thermo Scientific) and UV absorbance assay, respectively. Degree of degradation (%) = increased soluble protein (mg)/initial pig bristle weight (mg) × 100. Negative control: 2 × McIlvaine buffer (pH 8). The degree of degradation was calibrated to negative control.
